# Bile acid biosynthesis in Smith-Lemli-Opitz syndrome bypassing cholesterol: Potential importance of pathway intermediates

**DOI:** 10.1016/j.jsbmb.2020.105794

**Published:** 2021-02

**Authors:** Jonas Abdel-Khalik, Thomas Hearn, Alison L. Dickson, Peter J. Crick, Eylan Yutuc, Karl Austin-Muttitt, Brian W. Bigger, Andrew A. Morris, Cedric H. Shackleton, Peter T. Clayton, Takashi Iida, Ria Sircar, Rajat Rohatgi, Hanns-Ulrich Marschall, Jan Sjövall, Ingemar Björkhem, Jonathan G.L. Mullins, William J. Griffiths, Yuqin Wang

**Affiliations:** a Swansea University Medical School, ILS1 Building, Singleton Park, Swansea, SA2 8PP, Wales, UK; b Stem Cell & Neurotherapies, Faculty of Biology, Medicine and Health, University of Manchester, Manchester, M13 9PT, UK; c Willink Unit, Manchester Centre for Genomic Medicine, Manchester University Hospitals, Manchester, M13 9WL, UK; d University of California San Francisco (UCSF) Benioff Children’s Hospital, Oakland, CA 94609, USA; e Inborn Errors of Metabolism, Genetics and Genomic Medicine, UCL Great Ormond Street Institute of Child Health, 30 Guilford Street, London, WC1N 1EH, UK; f Department of Chemistry, College of Humanities & Sciences, Nihon University, Sakurajousui, Setagaya, Tokyo, 156-8550, Japan; g Departments of Biochemistry and Medicine, Stanford University School of Medicine, Stanford, CA, 94305, USA; h Department of Molecular and Clinical Medicine, University of Gothenburg, Sahlgrenska Academy, Institute of Medicine, Gothenburg, 41345, Sweden; i Department of Medical Biochemistry and Biophysics, Karolinska Institutet, Stockholm, 17177, Sweden; j Division of Clinical Chemistry, Department of Laboratory Medicine, Karolinska Institutet, Karolinska University Hospital Huddinge, Huddinge, 14186, Stockholm, Sweden

**Keywords:** SLOS, Smith-Lemli-Opitz syndrome, 7-DHC, 7-dehydrocholesterol, LC–MS, liquid chromatography – mass spectrometry, Smo, smoothened (FZD11), Hh, hedgehog, CYP, cytochrome P450, CYP7A1, cytochrome P450 family 7 subfamily A member 1, CYP27A1, cytochrome P450 family 27 subfamily A member 1, 26−HC, (25R)26-hydroxycholesterol, 27−HC, 27-hydroxycholesterol, CH25H, cholesterol 25-hydroxylase, CYP46A1, cytochrome P450 family 46 subfamily A member 1, FXR, farnasoid X receptor, LXR, liver X receptor, PXR, pregnane X receptor, VDR, vitamin D receptor, CAR, constitutive androstane receptor, GPCR, G protein-coupled receptor, GPR183, Epstein Barr virus induced gene 2 (EBI2), GPBAR1, G protein-coupled bile acid receptor 1, NMDAR, *N*-methyl-d-aspartate receptor, CSF, cerebrospinal fluid, CNS, central nervous system, 3β, 7α-diHCA, 3β,7α-dihydroxycholest-5-en-(25R)26-oic acid, CTX, cerebrotendinous xanthomatosis, DHCR7, 7-dehydrocholesterol reductase, 25H, 7O-C, 3β,25-dihydroxycholest-5-en-7-one, 7β, 25-diHC, 7β,25-dihydroxycholesterol, 26H, 7O-C, 3β,26-dihydroxycholest-5-en-7-one, 7β, 26-diHC, 7β,26-dihydroxycholesterol, 3βH, 7O-CA, 3β-hydroxy-7-oxocholest-5-en-(25R)26-oic acid, 3β, 7β-diHCA, 3β,7β-dihydroxycholest-5-en-26-oic, 3β, 7β,24-triHCA, 3β,7β,24-trihydroxycholest-5-en-26-oic, 3β, 7β,25-triHCA, 3β,7β,25-trihydroxycholest-5-en-26-oic, 3βH, 7O-Δ^5^-BA, 3β-hydroxy-7-oxochol-5-en-24-oic, 3β, 7β-diH-Δ^5^-BA, 3β,7β-dihydroxychol-5-en-24-oic, MS^n^, multistage fragmentation, GlcNAc, *N*-acetylglucosamine, NP-C, Niemann-Pick disease type C, DHCR24, 24-dehydrocholesterol reductase, HSD11B1, hydroxysteroid (11-beta) dehydrogenase 1 (short chain dehydrogenase/reductase family 26C member 1), HSD11B2, hydroxysteroid dehydrogenase (11-beta) 2 (short chain dehydrogenase/reductase family 9C, member 3), BACS, bile acyl CoA-synthetase (SLC27A5, solute carrier family 27 member 5), AMACR, alpha-methylacyl-CoA-racemase, ACOX2, peroxisomal acyl-coenzyme A oxidase 2, DBP, peroxisomal multifunctional enzyme type 2 or HSD17B4, SPCx, sterol carrier protein x or sterol carrier protein 2, ACOT, acyl-CoA thioesterase, UGT3A1, 7β-hydroxy bile acid UDP *N*-acetylglucosaminyl transferase, SULT2A1, sulfotransferase family 2A member 1, BAAT, bile acid-CoA:amino acid *N*-acyltransferase, CRD, cysteine rich domain, 7−OC, 7-oxocholesterol, 7β−HC, 7β-hydroxycholesterol, GP, Girard P, HSD3B7, 3β-Hydroxy-Δ^5^-C27-steroid oxidoreductase (short chain dehydrogenase/reductase family 11E, member 3), Ptch1, Patched-1, SHH, Sonic hedgehog, Gli1, GLI family zinc finger 1, 20S−HC, 20S-hydroxycholesterol, LD, linker domain, TMD, transmembrane domain, ECL3, extracellular loop 3, zSmo, zebrafish Smo, SDS, sodium dodecyl sulfate, DTT, dithiothreitol, DSX, DrugScore eXtended, Sterol, Oxysterol, Bile acid, 7-dehydrocholesterol, Smith-Lemli-Opitz syndrome, High-Performance liquid chromatography, Mass spectrometry, Hedgehog signalling pathway

## Abstract

Bile acids are the end products of cholesterol metabolism secreted into bile. They are essential for the absorption of lipids and lipid soluble compounds from the intestine. Here we have identified a series of unusual Δ^5^-unsaturated bile acids in plasma and urine of patients with Smith-Lemli-Opitz syndrome (SLOS), a defect in cholesterol biosynthesis resulting in elevated levels of 7-dehydrocholesterol (7-DHC), an immediate precursor of cholesterol. Using liquid chromatography – mass spectrometry (LC–MS) we have uncovered a pathway of bile acid biosynthesis in SLOS avoiding cholesterol starting with 7-DHC and proceeding through 7-oxo and 7β-hydroxy intermediates. This pathway also occurs to a minor extent in healthy humans, but elevated levels of pathway intermediates could be responsible for some of the features SLOS. The pathway is also active in SLOS affected pregnancies as revealed by analysis of amniotic fluid. Importantly, intermediates in the pathway, 25-hydroxy-7-oxocholesterol, (25R)26-hydroxy-7-oxocholesterol, 3β-hydroxy-7-oxocholest-5-en-(25R)26-oic acid and the analogous 7β-hydroxysterols are modulators of the activity of Smoothened (Smo), an oncoprotein that mediates Hedgehog (Hh) signalling across membranes during embryogenesis and in the regeneration of postembryonic tissue. Computational docking of the 7-oxo and 7β-hydroxy compounds to the extracellular cysteine rich domain of Smo reveals that they bind in the same groove as both 20S-hydroxycholesterol and cholesterol, known activators of the Hh pathway.

## Highlights

• New pathway of bile acid biosynthesis avoiding cholesterol.
• Mechanism for biosynthesis of 3β,7β-dihydroxy-5-ene bile acids.
• Biosynthesis of 3β-hydroxy-7-oxo-5-ene bile acids.
• Pathway intermediates bind to and activate Smo.
• A new link between SLOS and deranged Hh signalling.

## 1 Introduction

Bile acids are a large family of steroids possessing an acidic group on the side-chain [1]. The family can be considered to include both C_24_ acids containing the four-ring steroid skeleton with a 5-carbon side-chain and C_27_ acids with an 8-carbon side-chain attached to the steroid skeleton [2]. They are synthesised in the liver [3], but steps in their biosynthesis may also proceed extrahepatically [4], e.g. in brain [5]. Bile acids are synthesised predominantly via two pathways. The dominating pathway in human is the “neutral” or “normal” pathway which starts with 7α-hydroxylation of cholesterol by the hepatic cytochrome P450 (CYP) 7A1 enzyme [3]. The second pathway, known as the “acidic” pathway, starts with (25R)26-hydroxylation of cholesterol by CYP27A1 to give (25R)26-hydroxycholesterol (26−HC) either in the liver or extrahepatically [3,4,6,7]. Note, we use the systematic numbering system to describe (25R)26-hydroxylation of cholesterol according to IUPAC rules, however, much of the literature describes the resulting product as 27-hydroxycholesterol (27−HC) [8]. Unless stated otherwise (25R) stereochemistry is generally assumed. See Supplemental Tables for lists of common and systematic names of compounds analysed in this work. Other minor pathways begin with 25-hydroxylation of cholesterol by cholesterol 25-hydroxylase (CH25H) [3,4,9] in e.g. activated macrophages [10], or with 24S-hydroxylation of cholesterol by cholesterol 24S-hydroxylase (CYP46A1) in brain [3]. Many of the subsequent enzymes converting hydroxycholesterols to bile acids are operative in multiple pathways allowing metabolite crossing between pathways [4,7].

The major bile acids in human are cholic, chenodeoxycholic, deoxycholic and lithocholic acids. The latter two are derived from the former two by 7α-dehydroxylation. Ursodeoxycholic is also present in human but rarely as a major bile acid [11]. Bile acids are secreted in bile as glycine or taurine conjugates, or in the case of lithocholic acid as a 3-sulfate. Bile acids function in the intestine to aid absorption of lipids and are recycled to the liver via the enterohepatic system. As well as functioning as detergents in the intestine bile acids are also signalling molecules, regulating their own synthesis via interaction with the farnesoid X receptor (FXR, NR1H4) [12], while intermediates in their biosynthetic pathways from cholesterol are ligands to other nuclear receptors e.g. liver X receptors (LXRs, NR1H3, NR1H2) [13], pregnane X receptor (PXR, NR1I2) [14], vitamin D receptor (VDR, NR1I1) [15], constitutive androstane receptor (CAR, NR1I3) [16]; to G protein-coupled receptors (GPCRs) e.g. GPR183 also named EBI2 [17] and GPBAR1 also called TGR5 [18]; and are allosteric modulators of *N*-methyl-d-aspartate receptors (NMDARs) [19]. Interestingly, some steps in bile acid biosynthesis may occur in the nervous system and almost all of the acidic pathway intermediates from cholesterol to bile acids can be found in brain or cerebrospinal fluid (CSF) [5,20], and many of these intermediates can cross the blood brain barrier providing traffic in and out of the central nervous system (CNS) [21,22]. Cholic acid has been identified in rodent brain [23] and shown to act as a ligand to LXRs regulating the neurogenesis of red nucleus neurons [24], while the C_27_ bile acid 3β,7α-dihydroxycholest-5-en-(25R)26-oic acid (3β,7α-diHCA) has been shown to regulate the survival of motor neurons, again through interaction with LXRs [25].

Unsurprisingly, deficiency in enzymes of the bile acid biosynthesis pathways lead to disease [26], however, as a consequence of the redundancy provided by multiple pathways, often not to a total elimination of bile acid formation e.g. in cerebrotendinous xanthomatosis (CTX) where even though there is a deficiency of CYP27A1 some cholic acid formation is maintained [27]. Likewise, defects in cholesterol biosynthesis result in clinical disorders [28], however, it is unknown if there is sufficient metabolic redundancy for cholesterol to be bypassed and bile acid biosynthesis maintained by alternative metabolic pathways.

**Fig. 1:**
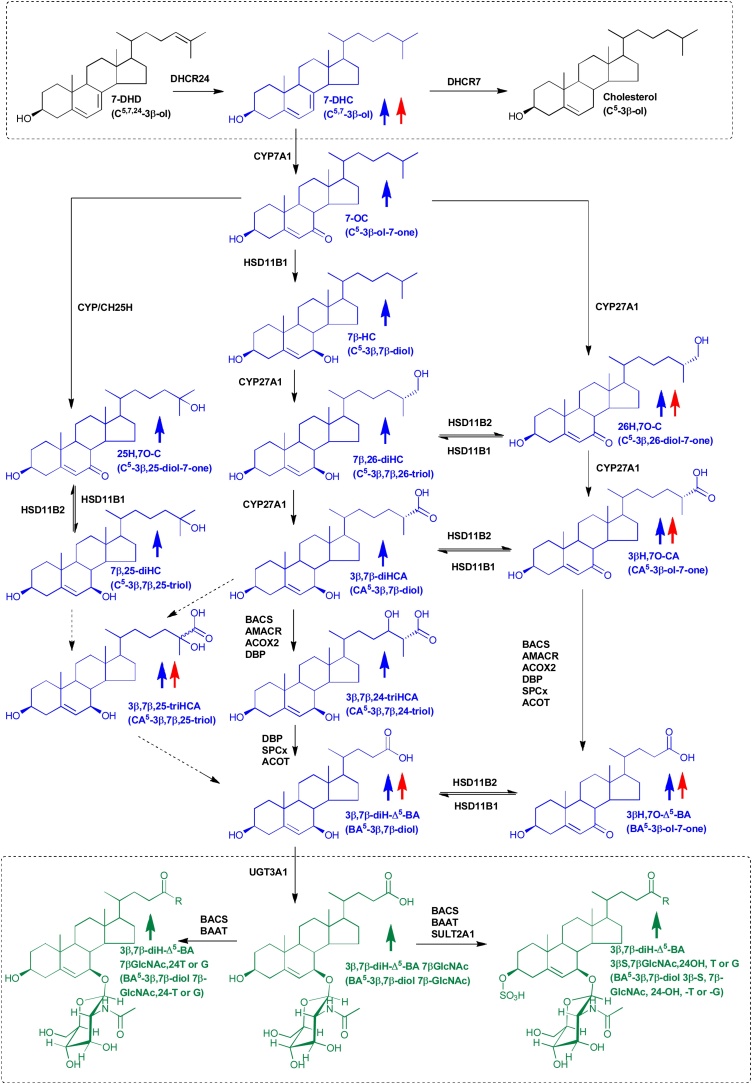
Bile acid biosynthesis starting with 7-DHC and ending with GlcNAc conjugates of 3β,7β-diH-Δ^5^-BA. The metabolites of elevated abundance found in plasma from SLOS patients are indicated by an upward pointing blue arrow and those elevated in amniotic fluid from SLOS affected pregnancies by an upward pointing red arrow. Metabolites of elevated abundance found in SLOS urine are indicated by a green upwards pointing arrow. Oxysterols, including C_24_ and C_27_ acids, found in plasma are shown in blue, bile acid conjugates found in urine are in green. The enzyme(s) for each step is in black. There is also minor non-enzymatic formation of 7−OC which may be more important in some lysosomal storage disorders [30]. Systematic and common names of molecular structures can be found in Supplemental Tables 1 & 2. For simplicity 3β,7β,24-triHCA is shown as the carboxylic acid rather than the CoA thioester. The 7-oxocholesterol 25-hydroxylase is not known, many CYP enzymes, and also CH25H, have sterol 25-hydroxylase activity.

In the current work we propose how uncommon Δ^5^-unsaturated bile acids can be biosynthesised via an unusual pathway from 7-dehydrocholesterol (7-DHC), an immediate precursor of cholesterol, in patients with the malformation disorder Smith-Lemli-Opitz syndrome (SLOS) where the enzyme 7-dehydrocholesterol reductase (DHCR7) is deficient and 7-DHC is abundant in tissues and plasma (Fig. 1). One branch of this pathway is also prevalent in SLOS-affected pregnancies as revealed here by analysis of amniotic fluid. We identify in human plasma the pathway intermediates 25-hydroxy-7-oxocholesterol (25H,7O-C), 7β,25-dihydroxycholesterol (7β,25-diHC), (25R)26-hydroxy-7-oxocholesterol (26H,7O-C), 7β,(25R)26-dihydroxycholesterol (7β,26-diHC), 3β-hydroxy-7-oxocholest-5-en-(25R)26-oic acid (3βH,7O-CA), 3β,7β-dihydroxycholest-5-en-(25R)26-oic acid (3β,7β-diHCA), 3β,7β,24-trihydroxycholest-5-en-26-oic acid (3β,7β,24-triHCA) and 3β,7β,25-trihydroxycholest-5-en-26-oic acid (3β,7β,25-triHCA) and the unsaturated C_24_ bile acids 3β-hydroxy-7-oxochol-5-enoic (3βH,7O-Δ^5^-BA) and 3β,7β-dihydroxychol-5-enoic acids (3β,7β-diH-Δ^5^-BA). With the exception of 3βH,7O-Δ^5^-BA, each of these metabolites was identified by liquid chromatography – mass spectrometry (LC–MS) with reference to authentic standards; 3βH,7O-Δ^5^-BA being identified by predicted accurate mass, retention time and multistage fragmentation (MS^n^) spectra. We did not have authentic standards of 3β,7β,24-triHCA or 3β,7β,25-triHCA but we did have standards for the 7α-epimers, the retention time and MS^3^ spectra of which give confidence to the identification of the 7β-epimers. The 7-oxo metabolites and also the ultimate metabolite, 3β,7β-diH-Δ^5^-BA, were also found in amniotic fluid from SLOS affected pregnancies. The Δ^5^-unsaturated C_24_ bile acids have previously been identified as their respective sulfate and *N-*acetylglucosamine (GlcNAc) conjugates in serum and urine from patients with the lysosomal storage disease Niemann-Pick disease type C (NP-C), as have also a number of these pathway intermediates [[29], [30], [31], [32]].

The biological significance of this unusual bile acid biosynthesis pathway found in SLOS and starting with 7-DHC is discussed in light of previous reports that pathway intermediates 25H,7O-C, 26H,7O-C and 7β,26-diHC bind to and activate the extracellular cysteine rich domain (CRD) of the oncoprotein Smoothend (Smo, FZD11) [33,34], a GPCR involved in the Hedgehog (Hh) signalling pathway which during embryogenesis controls the development of many tissues and after development regulates tissue stem cells and regenerative response to injury [35,36]. We show that 7β,25-diHC and other pathway intermediates, 3βH,7O-CA and 3β,7β-diHCA, also binds to Smo and activates Hh signalling. Through computational molecular docking we show that the 7-oxo compounds, 25H,7O-C, 26H,7−OC and 3βH,7O-CA, and the analogous 7β-hydroxysterols, bind to the same oxysterol binding groove in the CRD of Smo but show different patterns of hydrogen bonding. Importantly, abnormal Hh signalling has been implicated in human birth defects and many of the malformations found in SLOS are consistent with impaired Hh signalling [37].

## 2 Results

### 2.1 Plasma analysis

**Fig. 2:**
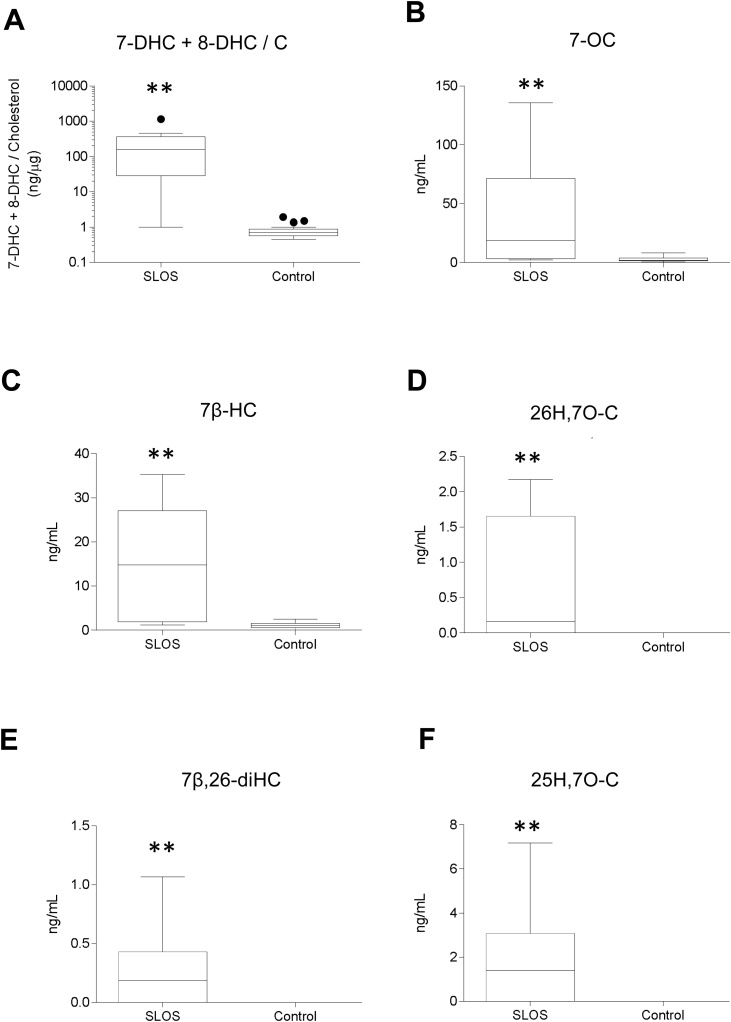
Concentration of 7-DHC and its metabolites in SLOS (n = 10 patients) and control (n = 24 volunteers) plasma samples. (A) Concentration of 7-DHC plus 8-DHC in ng/μg cholesterol. 8-DHC is an isomerisation product of 7-DHC. Concentrations of all other oxysterols are in ng/mL. (B) 7−OC. (C) 7β−HC. (D) 26H,7O-C. (E) 7β,26-diHC. (F) 25H,7O-C. Concentrations determined by LC–MS(MS^n^) exploiting charge-tagging utilising the Girard P reagent (GP) [42]. See Supplemental Figure S2 for relevant chromatograms. The bottom and top of the box are the first and third quartiles, and the band inside the box represents the median. The whiskers extend to the most extreme data points which are no more than 1.5 times the range between first and third quartile distant from the box. Points beyond that are plotted individually. Mann-Whitney test was used for comparison of non-normally distributed data. *, P < 0.05; **, P < 0.01. Data for 7-DHC + 8-DHC, 7−OC and 7β−HC has been reported in [44].

**Fig. 3:**
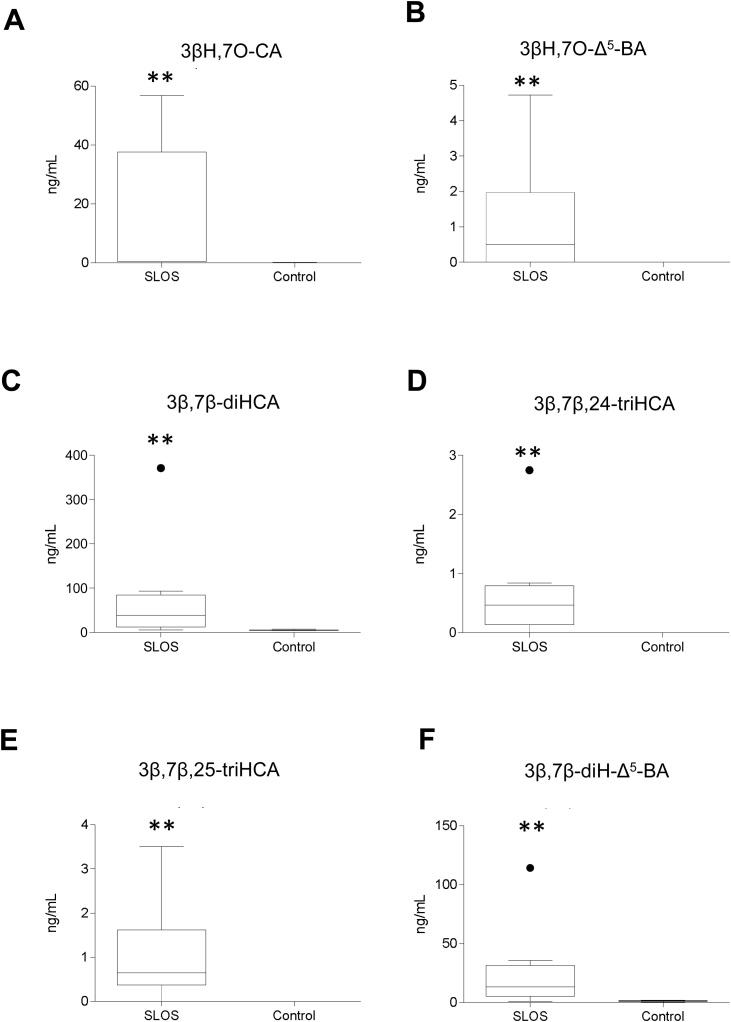
Concentration (ng/mL) of 7β-hydroxy and 7-oxo C_24_ and C_27_ acids in SLOS (n = 10) and control (n = 24) plasma. (A) 3βH,7O-CA. (B) 3βH,7O-Δ^5^-BA. (C) 3β,7β-diHCA. (D) 3β,7β,24-triHCA. (E) 3β,7β,25-triHCA. (F) 3β,7β-diH-Δ^5^-BA. See Supplemental Figure S2 for relevant chromatograms. Concentrations determined and statistical comparisons were as described in the caption to Fig. 2.

**Fig. 4:**
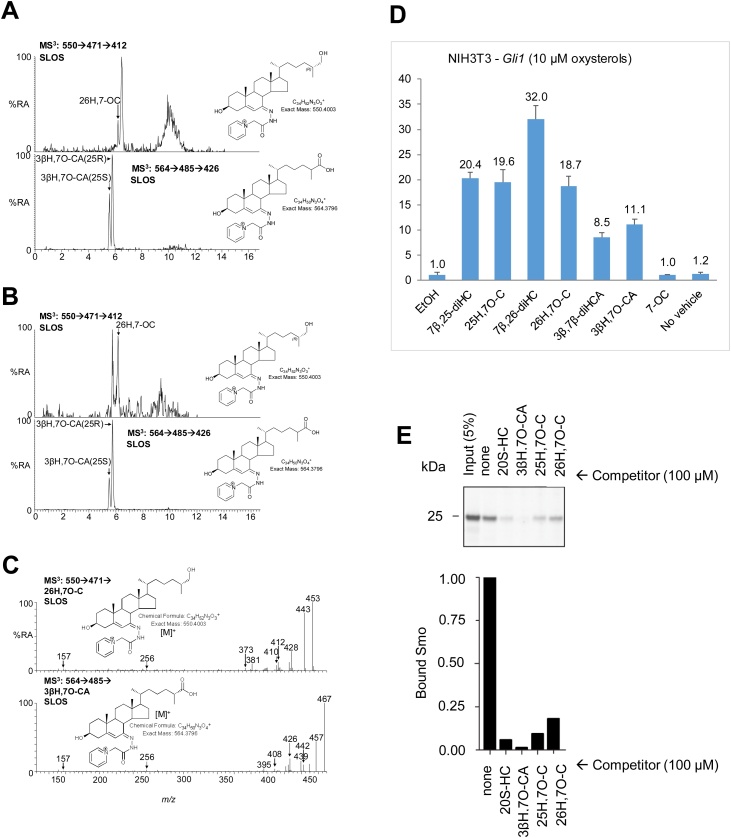
Identification of 7-oxo metabolites in SLOS plasma and amniotic fluid and demonstration that they modulated Hh signalling by binding to the CRD of vertebrate Smo. LC–MS^3^ chromatograms of GP-tagged 26H,7−OC (upper panel) and 3βH,7O-CA (lower panel) in SLOS (A) amniotic fluid; and (B) plasma. The identities of the sterols eluting after 26H,7−OC in the amniotic fluid sample and before 26H,7−OC in the plasma sample are unknown. SLOS samples are known to be rich in multiple sterols derived from free radical oxidation of 7-DHC, many of which are yet to be fully characterised [92]. LC–MS^3^ spectra from SLOS plasma identifying (C) 26H,7−OC (upper panel) and 3βH,7O-CA (lower panel). Spectra of authentic standards can be found in [93]. (D) Levels of *Gli1* mRNA (y-axis, mean arbitrary units ± SD, n = 3) were used as a metric for Hh signalling activity in NIH/3T3 cells after stimulation (7.5 h) with oxysterol (10 μM). With the exception of 3β,7β-diHCA the oxysterol agonists induced *Gli1* mRNA at 1 μM. (E) Immunoblots showing the amount of zSmo CRD captured on 20S−HC beads in the presence of the indicated competitors.

It has been shown that CYP7A1 can convert 7-DHC to 7-oxocholesterol (7−OC) [38] and it is known that 7−OC can be converted to 7β-hydroxycholesterol (7β−HC) by hydroxysteroid dehydrogenase (HSD) 11B1 in human and rodent (Fig. 1) [[39], [40], [41]]. This suggests that patients suffering from SLOS may use 7-DHC as a starting point for bile acid biosynthesis in addition to cholesterol. We thus investigated using LC–MS exploiting charge-tagging and MS^n^ (see Supplemental Figure S1) [30,[42], [43], [44]] the nature of bile acid intermediates found in plasma from patients suffering from SLOS. Previously, we were able to detect elevated levels of 7-DHC and its metabolites, 7−OC and 7β−HC (reported in (44)), and here we report elevated concentrations of the down-stream acids 3β,7β-diHCA and 3β,7β-diH-Δ^5^-BA in SLOS plasma (Figs. 2 & 3 , Supplemental Table S1, Supplemental Figure S2). Note, we did not include a saponification step in our sample preparation protocol, so concentrations of C_27_ sterols refer to non-esterified molecules. This is justified as it is the non-esterified sterols that have been identified to be biologically active. In patient samples where the 7-DHC + 8-DHC to cholesterol ratio is high (7-DHC isomerises to 8-DHC (44)), 26H,7O-C, 7β,26-diHC, 3βH,7O-CA and 3βH,7O-Δ^5^-BA acids were also observed (Fig. 1, Fig. 2, Fig. 3, Fig. 4, Supplemental Table S1, Supplemental Figure S2). In the absence of an authentic standard the latter compound was identified based on exact mass, MS^n^ spectra and retention time. Other compounds found to be elevated in SLOS samples where the 7-DHC + 8-DHC to cholesterol ratio is high were 25H,7O-C and 7β,25-diHC (Supplemental Table S1). Low levels of metabolites with retention time and MS^n^ fragmentation patterns consistent with 3β,7β,24-triHCA and 3β,7β,25-triHCA structures were presumptively identified by comparison to the 7α-epimers which were available as authentic standards (Fig. 3, Supplemental Table S1, Supplemental Figure S2). These twelve metabolites fall on three branches of an unusual bile acid biosynthesis pathway starting from 7-DHC and proceeding to 3β,7β-diH-Δ^5^-BA (Fig. 1).

As sterols with a 3β,7α-dihydroxy-5-ene structure are converted to analogous sterols with a 7α-hydroxy-3-oxo-4-ene structure in the neutral and acidic pathways of bile acid biosynthesis by the enzyme HSD3B7 [3], we investigated whether similar reactions proceeded with 3β,7β-dihydroxy-5-ene substrates. We found no evidence of sterols with a 7β-hydroxy-3-oxo-4-ene structure in our LC–MS analysis.

Unfortunately, we did not measure absolute concentrations of 7-DHC, 8-DHC or of cholesterol, only the 7-DHC + 8-DHC to cholesterol ratio [44]. Neither, do we have access to extensive clinical data, so we have not attempt to correlate metabolite levels to disease severity. However, it should be noted that the samples showing the highest 7-DHC + 8-DHC to cholesterol ratios also showed high concentration of pathway intermediates down-stream from 7−OC and 7β−HC, although not necessarily the highest concentrations *per se*. Looking in reverse, the SLOS plasma sample with the lowest 7-DHC + 8-DHC to cholesterol ratio in all cases showed the lowest levels of pathway intermediates beyond 7−OC and 7β−HC. It is of interest that the outliers Figs. 3C, D and F, relating to concentrations of 3β,7β-diHCA, 3β,7β,24-triHCA and 3β,7β-diH-Δ^5^-BA, the three final metabolites in the pathway from 7β−HC to 3β,7β-diH-Δ^5^-BA are all from the same sample. The level of 7β−HC in this sample was similar to the mean for the SLOS sample set, while levels of the 7-oxo metabolites in this sample were low. This data suggests high activity of HSD11B1 (reductase) compared to HSD11B2 (oxidase) in this patient. Further studies with well curated clinical information on a larger set of samples are planned.

### 2.2 Amniotic fluid analysis

Among other vital roles, amniotic fluid serves to facilitate the exchange of biochemicals between mother and foetus [45]. Previous studies have shown that SLOS affected-pregnancies can be diagnosed by MS analysis of this fluid through measuring elevated levels of 7-DHC and its isomer 8-DHC generated by the affected foetus [46,47]. As SLOS presents before birth it is possible that metabolites of the unusual pathway of bile acid biosynthesis may be present in amniotic fluid. As reported previously [47], we found an increase in the ratio of 7-DHC plus 8-DHC to cholesterol in SLOS affected-pregnancies. In addition, levels of 26H,7O-C, 3βH,7O-CA, 3βH,7O-Δ^5^-BA, 3β,7β,25-triHCA and 3β,7β-diH-Δ^5^-BA were similarly elevated in SLOS amniotic fluid samples (Supplemental Table S1, Supplemental Figure S3).

### 2.3 Urine analysis

**Fig. 5:**
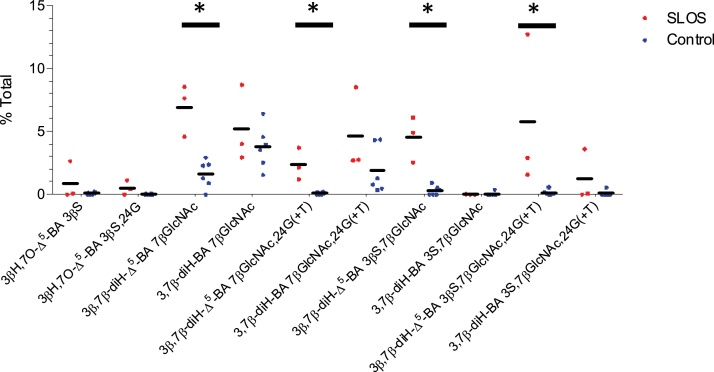
Relative proportions (mole %) of bile acids with 7-oxo or 7β-hydroxy group conjugated with GlcNAc in urine from 3 SLOS patients and 6 controls determined by LC–MS. The SLOS patients were from the group who donated plasma. See Supplemental Figure S4 for relevant chromatograms. Total bile acids include mono-, di- and tri-hydroxylated cholanoic acids and their single and doubly unsaturated equivalents singly, doubly or triply conjugated with glycine or taurine, sulfuric acid and GlcNAc. Mann-Whitney test was used for comparison of non-normally distributed data. *, P < 0.05; **, P < 0.01.

Sterols with a 3β,7β-dihydroxy-5-ene function have not been reported to be substrates for HSD3B7 [48], the oxidoreductase normally required to initiate A/B ring transformation ultimately leading to the 3α-hydroxy-5β-hydrogen configuration found in primary bile acids [3]. We failed to detect any 7β-hydroxy-3-oxo-4-ene sterols in our analysis of SLOS plasma, supporting the data of Furster et al. and the contention that HSD3B7 is inactive towards 3β,7β-dihydroxy-5-ene sterols, at least in human and pig [48]. We propose that the 3β,7β-dihydroxy-5-ene structure is maintained in the pathway intermediates from 7-DHC to bile acids as described by the central branch of the pathway in Fig. 1. Sterols possessing a 7β-hydroxy group are known to be conjugated with GlcNAc and excreted in urine [49], hence it might be expected that the urine of SLOS patients would contain elevated levels of unsaturated GlcNAc conjugated bile acids. Using LC—MS at high mass resolution with MS^n^, i.e. LC–MS(MS^n^), we determined the total bile acid content of urine by summing the amounts of mono-, di- and trihydroxycholanoic acids, their single and doubly unsaturated equivalents and single, double and triple conjugates with glycine or taurine, sulfuric acid and GlcNAc. We found significantly elevated levels of 3β,7β-diH-Δ^5^-BA conjugated with GlcNAc at position 7β in urine from SLOS patients and also the double conjugate with sulfuric acid (S) at C-3β (Fig. 5). Note, that we did not have authentic standards of the Δ^5^-unsaturated GlcNAc conjugates, but LC-retention times, exact mass measurements and MS^2^ spectra were consistent with 7β-GlcNAc conjugation. While we are confident that the sugar is an *N-*acetylhexosamine (HexNAc), based on accurate mass and MS^2^ spectra, we cannot be absolutely sure that it is GlcNAc (in the absence of GC–MS analysis of the monosaccharide), however, to the best of our knowledge no other HexNAc sugar has been found conjugated to a bile acid at C-7 [1,11,29,49]. Other double conjugates with GlcNAc (C-7β) and also glycine or taurine (C-24), and triple conjugates with glycine or taurine, sulfuric acid and GlcNAc were also found to be elevated significantly in SLOS urine (Figs. 1 & 5, Supplemental Table S2, Supplemental Figure S4). There was no overlap in the levels of the 3β,7β-diH-Δ^5^-BA GlcNAc conjugates in urine samples from the SLOS and control groups, although we did only analyse three SLOS samples and six control samples. We also found elevated levels of 3βH,7O-Δ^5^-BA conjugated with sulfuric acid in one of the patient samples analysed and increased levels of the double conjugate with sulfuric acid and glycine in two patient samples.

### 2.4 Hedgehog signalling

**Fig. 6:**
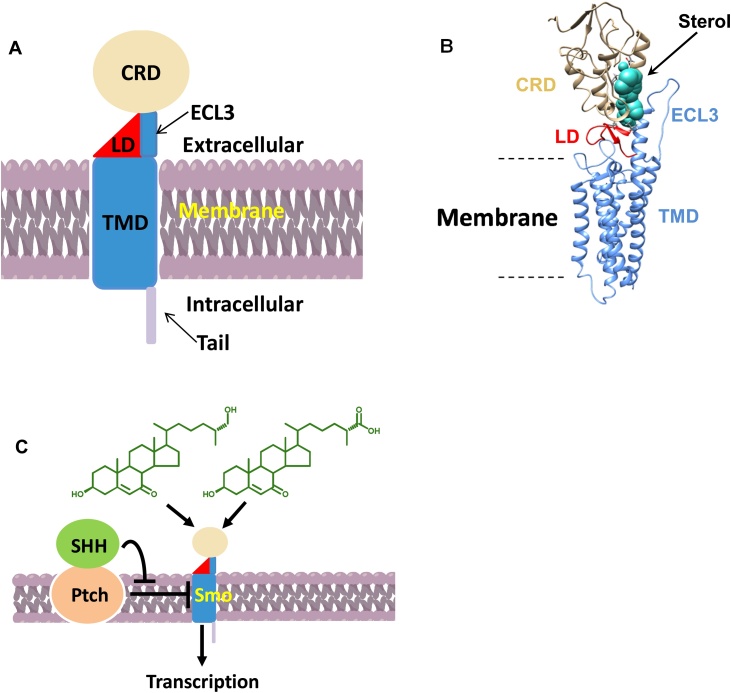
Oxysterols bind to Smo and modulate Hh signalling across the cell membrane. (A) Cartoon representation of Smo (CRD in silver, LD in red, TMD including ECL3 in blue. (B) Molecular modeling analysis of human Smo with cholesterol bound to the CRD, based on the crystal structure in [52]. (C) Schematic representation of the Hh signalling pathway showing 7-oxosterols activating Smo.

Hh signalling provides a cell – cell communication system across membranes in all animals. The receptor for the Hh-protein ligands, Patched 1 (Ptch1) inhibits signalling by supressing the activity of Smo (Fig. 6). Sonic hedgehog (SHH) protein binds to and inhibits Ptch1, allowing Smo to adopt an active conformation and transmit signal across the plasma membrane, ultimately leading to nuclear translocation of the Gli family of Hh transcription factors and activation of Hh target genes including *Gli1* [36,50]. Recent studies have indicated that 25H,7O-C, 26H,7O-C and 7β,26-diHC, can activate the Hh signalling pathway in the absence of SHH [33,34]. Thus, we performed a Hh signalling assays using quantitative RT-PCR to measure mRNA levels of *Gli1*, commonly used as a metric for Hh signalling strength, following stimulation (7.5 h) of NIH/3T3 cells with these three oxysterol and other oxysterols found to be elevated in SLOS plasma. As expected, 25H,7O-C, 26H,7O-C and 7β,26-diHC induced *Gli1* mRNA, as did other pathway metabolites 7β,25-diHC, 3βH,7O-CA and 3β,7β-diHCA (Fig. 4). For comparison incubation of NIH/3T3 cells with 300 nM SHH gave about the same level of *Gli1* mRNA as 10 μM 26H,7O-C.

As SLOS is a developmental disorder, we decided to focus on the 7-oxo branch of the unusual pathway in Fig. 1 which involves metabolites found to be elevated in amniotic fluid from SLOS affected pregnancies. To investigate if the 7-oxo compounds bind to Smo through its extracellular CRD we used the purified CRD from zebrafish Smo and tested it for binding to 20S-hydroxycholesterol (20S−HC) immobilised on beads [35] in the presence of the 7-oxo competitors. 20S−HC is known to bind to the CRD of vertebrate Smo and to activate Hh signalling [35,51]. In the absence of competitor (none) Smo robustly binds to 20S−HC beads (Fig. 4) [35,51]. In agreement with an earlier report, 26H,7O-C and 25H,7O-C are very good competitors for Smo binding [33], similar to 20S−HC. We now find that 3βH,7O-CA also acts as a competitor for the binding of Smo CRD to 20S-beads (Fig. 4). These data provide support for the concept that the 7-oxo compounds can bind to the extracellular CRD of Smo and modulate Hh signalling during development. As SLOS is a disease which phenocopies deficient Hh signalling [37], dysregulation of the balance between SMO modulators may be an explanation for this deficiency and consequently of malformations seen in SLOS.

### 2.5 Molecular docking

**Fig. 7:**
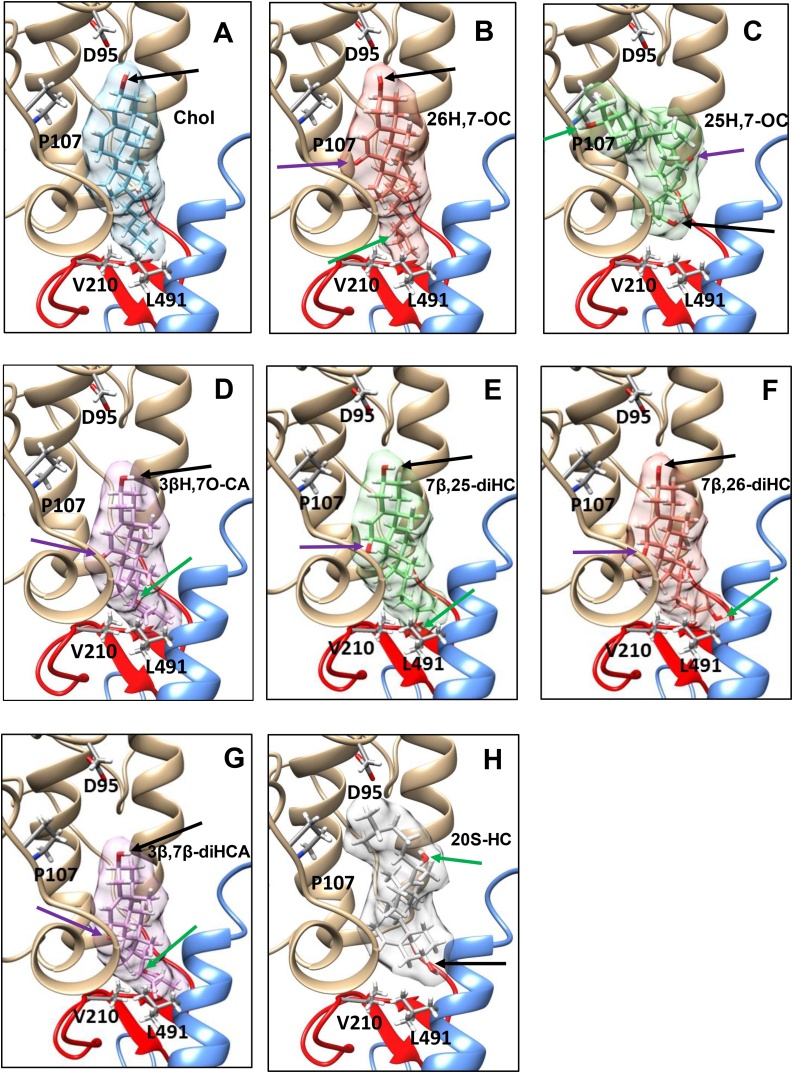
Computationally docked structures of (A) cholesterol; (B) 26H,7O-C; (C) 25H,7O-C, (D) 3βH,7O-CA; (E) 7β,25-diHC; (F) 7β,26-diHC; (G) 3β,7β-diHC; and (H) 20S−HC bound to the CRD of human Smo. In silico protein-ligand docking studies were performed using a combination of empirical and force-field approaches incorporated within an in-house pipeline called “Shipyard”, to predict the conformation and affinity of binding. 3β-hydroxy; 7β-hydroxy or 7-oxo; and side-chain hydroxy or carboxylic acids; are indicated by black; purple; and green; arrows respectively.

Recently, Byrne et al. solved the crystal structure of the human Smo protein consisting of the CRD (silver in Figs. 6 & 7 ), linker domain (LD, red) and the entire transmembrane domain (TMD, blue) but lacking its cytoplasmic tail, and unexpectedly found a cholesterol molecule bound to the CRD [52]. They concluded that Smo adopts an extended conformation, with the extracellular CRD perched on top of the LD, which forms a wedge between the TMD and CRD. At the top of the wedge the CRD contacts the TMD through extracellular loop 3 (ECL3) of the TMD (Fig. 6). To investigate how the 7-oxo compounds which modulate Hh signalling and are found in SLOS plasma and in amniotic fluid, interact with Smo we carried out computational ligand docking [53] of these and other sterols to the human Smo CRD. The results show accurate docking of cholesterol (Fig. 7A) in the same location and almost identical orientation in the CRD groove as in the cholesterol-bound crystal structure [52], with good affinity, with the 3β-hydroxy group and sterol rings interacting with Asp95 (D95) and Pro107 (P107), respectively, of the CRD domain, with the sterol side-chain pointing downwards towards Leu491 (L491) of the helical region of ECL3 and Val210 (V210) of the LD region. The docking of 26H,7O-C (Fig. 7B) is almost identical to that of cholesterol. The compound occupies the same pocket in almost the same orientation with only sub-Ångstrom differences. The predicted binding affinity is slightly lower than for cholesterol (0.689 kcal/mol). Notably, Luchetti et al. have shown that, like 26H,7O-C, cholesterol activates the Hh signalling pathway by binding to the extracellular CRD of Smo [54]. 25H,7O-C binds to the CRD with a notably different orientation, with the 3β-hydroxy group and sterol rings pointing downwards towards the helical region of ECL3 and the side-chain, including 25-hydroxy group, pointing upward (Fig. 7C). Binding affinity is calculated to be 0.936 and 0.247 kcal/mol weaker than for cholesterol and 26H,7O-C, respectively, but is still reasonably strong. There is no projection towards the ECL3 or LD regions. 3βH,7O-CA binds with comparable affinity to cholesterol, in the same pocket, and with a similar broad orientation, with good LD region proximity (V210) via the side-chain but slightly closer to the helical region of ECL3 (L491) than cholesterol, but with the 3β-hydroxy group and sterol rings further away from D95 and P107, respectively (Fig. 7D). The H-bond of the 3β-hydroxy group to D95 found in 26H,7−OC and cholesterol is lost in the 3βH,7O-CA bound structure. 7β,25-diHC, 7β,26-diHC, and 3β,7β-diHCA bind more deeply in the pocket (Fig. 7E-G), in similar orientation to 3βH,7O-CA, with side-chain proximity to the LD region at V210 and to the helical region of ECL3 (L491), but with greater affinity than 3βH,7O-CA and similar affinity to cholesterol. Indeed, the calculated binding affinities of 7β,25-diHC and 3β,7β-diHCA are marginally stronger than for cholesterol by 0.134 and 0.218 kcal/mol, respectively. Interestingly, 3β,7β-diHCA, with the greatest affinity, sits deepest in the pocket. In agreement with a previously reported docking [35], 20S−HC binds to the same groove as cholesterol but in the opposite orientation (Fig. 7H, cf. H to A), with the sterol side-chain pointing towards D95. Despite this both cholesterol and 20S−HC activate Hh signalling via the same CRD pocket [54].

## 3 Discussion

Bile acid biosynthesis normally starts from cholesterol, however, CYP7A1 can also use 7-DHC as a substrate giving 7−OC as a product [38] which can be reduced by HSD11B1 to 7β−HC [[39], [40], [41]] opening a new route to bile acid biosynthesis, starting from 7-DHC and avoiding cholesterol (Fig. 1). The elevated levels of these two oxysterols in plasma of SLOS patients [44] and also those of 3β,7β-diHCA and 3β,7β-diH-Δ^5^-BA define a new and unexpected pathway for bile acid biosynthesis in SLOS patients (Fig. 1, Fig. 2, Fig. 3). Further evidence for this pathway is provided by the presumptive identification of 3β,7β,24-triHCA, a necessary intermediate as the CoA thioester in peroxisomal side-chain shortening of 3β,7β-diHCA to 3β,7β-diH-Δ^5^-BA (Fig. 1). Although the identification of 3β,7β,24-triHCA, and that of 3β,7β,25-triHCA, was made in the absence of authentic standards, the availability of standards for the 7α-epimers allowed the confident identification of 7β-epimers based on accurate mass, expected retention times and MS^3^ spectra (Supplemental Figure S2I - N). A second branch of the pathway is initiated by CYP27A1 mediated (25R)26-hydroxylation of 7−OC [55] and defined by the identification of 26H,7O-C, 3βH,7O-CA and 3βH,7O-Δ^5^-BA in SLOS plasma from patients with a high 7-DHC plus 8-DHC to cholesterol ratio. Each 7-oxo compound presumably acts as a substrate for HSD11B1 and can be reduced to the 7β-hydroxy analogue [56]. A third branch to the pathway may proceed through 25H,7O-C, 7β,25-diHC and 3β,7β,25-triHCA, although it is not known whether CYP27A1 is responsible for the oxidation of the terminal carbon to the carboxylic acid and how the resulting acidic triol undergoes side-chain shortening (Fig. 1). The initiating enzyme of this third branch is not known as numerous CYP enzymes have 25-hydroxylase activity, as does CH25H [57]. Odermatt and colleagues have shown that both 25H,7O-C and 26H,7O-C can be reduced to their 7β-hydroxy analogues by HSD11B1, while the reverse reaction can be catalysed by HSD11B2 [56,58], and we speculate that 7-oxo and 7β-hydroxy acids may be similarly interconverted by these two enzymes (Fig. 1).

As SLOS is a developmental disorder [37,59], we hypothesised that the unusual bile acid biosynthesis pathway may be active in the foetus and responsible for some of the phenotypical features of the disorder. To test the first part of this hypothesis we analysed amniotic fluid from SLOS affected pregnancies where both parents were carriers of SLOS (heterozygous with respect to mutations in *DHCR7*). As reported previously [47], 7-DHC plus 8-DHC was elevated in SLOS affected pregnancies and, as is detailed here, so are the ultimate unconjugated bile acids, 3β,7β-diH-Δ^5^-BA and 3βH,7O-Δ^5^-BA, in the pathway depicted in Fig. 1. The first enzyme in this pathway, required to convert 7-DHC to 7−OC, is microsomal CYP7A1, this is expressed in adult liver [3], but in the infant is normally of only minor importance in bile acid synthesis [60]. However, microsomal 7α-hydroxylase activity is found in foetal liver [61,62] and CYP7A1 is evident in foetal heart and testis [63]. Mitochondrial CYP27A1 activity has been found in foetal liver [64], providing a route for (25R)26-hydroxylation and carboxylation of 7−OC [55,65], and also presumably of 7β−HC. HSD11B1, the enzyme required to reduce 7-oxosterols to their 7β-hydroxy analogues, is highly expressed in placenta, as is HSD11B2, the enzyme that catalyses the reverse reaction [63,66]. This suggests a route to the 7β-hydroxy branch of the unusual pathway and cross over between different branches of the pathway via placenta and umbilical cord blood. The fact that others have found primary C_24_ bile acids in foetal gall bladder bile confirms that the foetus possesses the enzymatic machinery for side-chain shortening of C_27_ precursors to C_24_ acids [67,68], indicating that the necessary enzymes are present in the foetus to generate both 3β,7β-diH-Δ^5^-BA and 3βH,7O-Δ^5^-BA (Fig. 1).

The 7β-hydroxy group in bile acids is known to become conjugated with GlcNAc, leading to the excretion of GlcNAc conjugates in urine [49]. Screening for bile acids in urine of SLOS patients to confirm the validity of the unusual pathway revealed statistically significant elevated levels of 3β,7β-diH-Δ^5^-BA conjugated with GlcNAc at the 7β position and also the double conjugate with glycine or taurine or with sulfuric acid, and the triple conjugate with GlcNAc, sulfuric acid and glycine or taurine (Fig. 5). The levels of these GlcNAc conjugates in SLOS urine do not overlap with levels of the corresponding compounds in control urine.

Although in this study we have made no attempt to correlate SLOS severity with 7-oxo, 7β-hydroxy or 7β-GlcNAc metabolite levels the possibility exists that some of these metabolites may be of diagnostic or prognostic value. Furthermore, the identification of GlcNAc or sulfuric acid conjugates in maternal urine could have value in prenatal screening programmes, although these metabolites are also identified in NP-C patients [29,31,32].

The data presented above is supported by a study performed on SLOS urine by Natowicz and Evans in 1994 using fast atom bombardment MS [69]. Although in the absence of chromatography or MS^2^ they were unable to fully characterise the metabolites, they were able to identify hydroxyoxocholenoic (H,O-Δ-BA) and dihydroxycholenoic (diH-Δ-BA) acids, their sulfuric acid conjugates and also a dihydroxycholenoic acid doubly conjugated with sulfuric acid and an aminohexose sugar [69]. Based on our studies these bile acid structures are appropriate to 3βH,7O-Δ^5^-BA and 3β,7β-diH-Δ^5^-BA and their conjugates with sulfuric acid and GlcNAc.

Others have also studied bile acids in relation to SLOS [70,71]. Using a rat model for SLOS Honda et al. identified unusual Δ^5,7^ and Δ^5,8^ 3β-hydroxycholestadienoic acids from rat liver mitochondrial preparations, along with their precursors 26-hydroxy-7-dehydrocholesterol and 26-hydroxy-8-dehydrocholesterol [70]. The identification of the latter metabolite in SLOS plasma was confirmed in a subsequent report [44]. Unlike in the present study, Honda et al. did not profile for 7-oxo or 7β-hydroxy acids. Steiner et al. have analysed free bile acids from stool of SLOS patients and controls after cleavage of conjugates but did not structurally identify the unusual bile acids present in the samples [71].

Shoda et al. have suggested that enzymatic mitochondrial oxidoreduction of 7α-hydroxy-5-ene sterols to their 7β-hydroxy epimers can occur in liver [72]. In fact, they found that human liver mitochondria rapidly convert 7α,26-diHC and 3β,7α-diHCA to their corresponding 7β-hydroxy epimers in an isocitrate dependent manner, perhaps through their 7-oxo intermediates, providing an alternative route to 3β,7β-diH-Δ^5^-BA [72]. Additionally, 7α-hydroxy epimerisation may be catalysed by the intestinal flora during the enterohepatic circulation to produce ursodeoxycholic and ursocholic acids present in bile and urine of healthy humans [49,73]. It is likely, that there are several pathways to 7β-hydroxy bile acids in human of varying importance in health and disease.

It is noteworthy that previous studies have identified 3β,7β-diH-Δ^5^-BA singly conjugated with GlcNAc at the C-7β position, doubly conjugated with GlcNAc and sulfuric acid at C-7β and C-3β, respectively, and triply conjugated with GlcNAc, sulfuric acid and glycine or taurine at C-7β, C-3β and C-24, respectively, in urine of patients suffering from NP-C disease [29,31,32]. NP-C is a rare inherited lipid trafficking disorder which amongst other symptoms presents with learning difficulties and progressive intellectual decline, behaving as a neurodegenerative disorder. In contrast, SLOS causes microcephaly and developmental delay. Children with NP-C do not show the classic dysmorphic features seen in SLOS. In one of the early studies on NP-C, 3βH,7O-Δ^5^-BA was identified as the 3β-sulfate and also conjugated with glycine or taurine and it was suggested that 3βH,7O-Δ^5^-BA was an intermediate in the biosynthesis of the 3β,7β-diH-Δ^5^-BA 7β-GlcNAc conjugates [29]. In a latter study both 3β,7β-diH-Δ^5^-BA and 3βH,7O-Δ^5^-BA were identified in NP-C and NP-B plasma [30]. Recent studies by Maekawa et al. suggested 3β,7β-diH-Δ^5^-BA conjugated with sulfuric acid at C-3β and GlcNAc at C-7β as a urinary biomarker for NP-C disease [32]. However, Clayton and colleagues have found that some NP-C patients carry the cT361G mutation in *UGT3A1* leading to the amino acid substitution p.C121G and an absence of activity of the encoded 7β-hydroxy bile acid UDP *N*-acetylglucosaminyl transferase [31]. As 20 % of the Asian and Caucasian populations are homozygous for this mutation, it can be expected that 20 % of NP-C and also of SLOS patients will not show GlcNAc conjugates in urine. In the absence of functional 7β-hydroxy bile acid UDP *N*-acetylglucosaminyl transferase it can be expected that sulfuric acid conjugates of 7β-hydroxy- and 7-oxo-Δ^5^-bile acids would be further elevated in urine. In our small study of urine samples, all SLOS patients excreted GlcNAc conjugates, so this hypothesis was not tested. In the original publication by Alvelius et al. it was suggested that the 7-oxo- and 7β-hydroxy bile acids observed may be a consequence of extensive lipid peroxidation of cholesterol in NP-C disease [29]. This seems likely to be correct as elevated levels of 7−OC and also cholestane-3β,5α,6β-triol, derived enzymatically from 5,6-epoxycholesterol, like 7−OC a peroxidation products of cholesterol, are found in plasma of NP-C patients [30,31]. Importantly, cholestane-3β,5α,6β-triol is not elevated in plasma of SLOS patients indicating that different oxidative mechanisms are at play in SLOS and NP-C. Additionally, the absence or presence of elevated levels cholestane-3β,5α,6β-triol or its further metabolites in plasma allows the simple biochemical differentiation of SLOS from NP-C. The presence of elevated concentrations of 7−OC in NP-C plasma, in this case generated through peroxidation mechanisms, should also lead to a pattern of bile acid precursors similar to those presented in Fig. 1. This is in fact the case and supports the bile acid biosynthesis pathway presented in Fig. 1 [30].

The SLOS phenotype is very broad; severely affected cases often die *in utero* or soon after birth, whereas mild cases show only minor physical abnormalities and learning and behavioural problems [37]. Limb abnormalities are common in SLOS and, in addition to physical malformations, SLOS patients have impaired cognitive function although normal intelligence is also possible [37]. Some SLOS patients present with cholestatic liver disease, as do some patients with the bile acid synthetic defect CTX and larger numbers of patients with NP-C disease, perhaps due to a lack of primary bile acid dependent bile flow, as in the primary defects of bile acid biosynthesis, but this could also be due to potential toxicity of abnormal bile acids. Although the primary enzymatic defect in SLOS is well defined, its pathophysiology is not, and it is unlikely that only one mechanism explains the myriad of symptoms. It is tempting to speculate that some of the metabolites of the newly defined bile acid biosynthesis pathway (Fig. 1) explain some of SLOS phenotypic features. In fact, 3β,7β-diHCA is toxic towards neurons and may be responsible for some of the neurological symptoms of the disease [25].

Hh signalling is required for embryonic patterning and regeneration of postembryonic tissue and aberrant Hh signalling has been linked to SLOS [33,37,[74], [75], [76]]. In fact, many developmental malformations attributed to SLOS occur in tissues where Hh signalling is required for development [75]. DHCR7, the defective enzyme in SLOS, has been implicated to function as a positive regulator of Hh signalling, while the cause of some of the developmental abnormalities seen in SLOS have been attributed to cholesterol deficiency interfering with normal Hh signalling [74,76]. Alternatively, Koide et al. have suggested that DHCR7 functions as a negative regulator of Hh signalling and its inhibitory affect is at the level, or downstream, of the oncoprotein Smo [75]. Both of these proposals can be accommodated by a model suggested by Beachy and colleagues where oxysterols and cholesterol could bind to and modulate Smo at different structural regions [33,77]. Smo is a seven-transmembrane protein with extended extracellular and cytoplasmic termini. Hh pathway activation is initiated by binding of cholesterol-modified SHH protein to its receptor Ptch1, an antiporter protein, which releases inhibition of Smo and triggers transcription of Hh target genes via the Gli family of transcription factors (Fig. 6) [36,78,79]. It has been shown that the extracellular CRD is the site for oxysterol binding to Smo and suggested that oxysterols may stabilise an active Smo conformation [33,51,80]. Recently, Byrne et al. determined the crystal structure of Smo and found a cholesterol molecule bound to the CRD [52]. They proposed that cholesterol functions as an endogenous Smo ligand that occupies the CRD groove and there is now compelling evidence that cholesterol is sufficient to activate Hh signalling through the CRD site [54,81,82]. Interestingly, Deshpande et al. have shown that when stabilised in an active state, SMO has two sterol binding sites, one in its CRD and a second in the transmembrane domain [77], however, most evidence to date indicates that activating oxysterols bind to the CRD. With respect to Hh signalling, 20S−HC has been regarded as an archetypal oxysterol and has been shown to be a potent activator of Smo *in vitro* [51] but has been difficult to detect *in vivo*, questioning it physiological relevance [20,83,84]. However, recent studies have confirmed the earlier identification of 20S−HC in brain and in placenta [20,84]. It is difficult to compare the activation capacity of cholesterol with oxysterols, as while synthetic oxysterols can be added to cells, all cellular systems will be rich in endogenous cholesterol making effects due to addition of exogenous cholesterol difficult to assess [82]. Such comparisons are further complicated by the insolubility of cholesterol in cell media. Perhaps with the recent introduction of quantitative mass spectrometry imaging technology [20,85], relative levels of endogenous oxysterols and cholesterol can be measured in developing tissue e.g. developing vertebrate spinal cord.

In the current study, we identify 25H,7O-C and 26H,7O-C in SLOS plasma and confirm them, as reported earlier [33], as activators of Smo even in the absence of SHH (Fig. 4D). 26H,7O-C has been shown previously to be generated from 7−OC in HepG2 cells by CYP27A1 [55] (Fig. 1) and has also been identified in extracts of retinal pigment epithelial cells [65]. The enzyme involved in 25-hydroxylation of 7−OC to 25H,7−OC is less clear cut as multiple CYPs, and also CH25H, have 25-hydroxylase activity [3,4,57]. As 7−OC is derived from 7-DHC by CYP7A1 oxidation, the identification in the current study of SMO agonists 25H,7O-C in SLOS plasma and of 26H,7O-C in SLOS plasma and in amniotic fluid from SLOS affected pregnancies lends weight to the hypothesis of Koide et al. that DHCR7, which reduces the pool of 7-DHC substrate by metabolism to cholesterol (Fig. 1), functions as a negative regulator of Hh signalling at the level of Smo [75]. Although 25H,7O-C and 26H,7O-C are absent, or present at only very low levels, in control plasma and amniotic fluid samples the presence of down-stream metabolites in plasma and amniotic fluid from healthy individuals and pregnancies indicates that the pathway involving their formation is active in human. Like 26H,7O-C, 3βH,7O-CA has been identified in retinal pigment epithelial cells and is derived by CYP27A1 oxidation of 7−OC [65]. This acid which is present in SLOS plasma and amniotic fluid from affected pregnancies and to a minor extent in control samples, is structurally similar to 26H,7O-C and, as shown here, also activates Hh signalling (Fig. 4). The involvement of three 7-oxo metabolites of 7-DHC (i.e. 25H,7O-C, 26H,7O-C and 3βH,7O-CA) in Hh signalling can explain conflicting results suggesting DHCR7 is both a positive [74] and negative [75] regulator if one assumes that these 7-oxosterols have differing degrees of activity towards SMO and may act as partial agonists providing positive regulation, or competing against full agonists resulting in negative regulation. Although not detected in amniotic fluid, we show here that 7β,26-diHC and 7β,25-diHC both activate the Hh signalling pathway (Fig. 4D). This confirms the data of Raleigh et al. who also found that 7β,26-diHC activates the Hh signalling pathway via the CRD domain of SMO [34].

Our data from computational molecular docking of the 7-oxo and 7β-hydroxy compounds to the CRD of human Smo indicate binding to the same groove as cholesterol and 20S−HC but with each 7-oxidised metabolites in a somewhat different orientation (Fig. 7). The docking of 26H,7O-C is almost identical to that of cholesterol (Fig. 7A & B), but that of 25H,7O-C (Fig. 7C) is notably different with the 3β-hydroxy group and sterol rings pointing downwards towards the helical region of the ECL3 (blue in Fig. 7), rather than associating with the D95 loop and alpha helices of CRD (silver in Fig. 7), as is the situation with cholesterol and 26H,7O-C (Fig. 7A & B). The 25H,7O-C docking shows the sterol side-chain and 25-hydroxy group pointing upward and proximal to the P107 loop region (Fig. 7C). 3βH,7O-CA (Fig. 7D), binds more strongly to Smo than the agonists 26H,7O-C, 25H,7O-C or 20S−HC, in a similar orientation to 26H,7O-C but proximity to D95 towards the top of the binding groove is lost. 7β,25-diHC, 7β,26-diHC and 3β,7β-diHCA (Figure E—G) bind more deeply in the pocket, in similar orientation to 3βH,7O-CA with side-chain proximity to the LD region at V210 (red in Fig. 7) and to the helical region of ECL3 (L491), but with greater affinity than 3βH,7O-CA and similar affinity to cholesterol. Byrne et al. suggested that CRD agonists may induce a conformational change in Smo involving a shift of the ECL3 and pivoting of the CRD on the end of the TMD to provide signal transduction across the membrane.

The balance between accessible cholesterol, oxysterol agonists and of 3β,5α-dihydroxycholest-7-en-6-one, also derived from 7-DHC and recently shown to inhibit Hh signalling [86], may explain the broad spectrum of the SLOS phenotype. Interestingly, 3β,5α-dihydroxycholest-7-en-6-one, which we previously found in SLOS plasma at about the same level as reported here for 26H,7O-C [44], inhibits the agonistic effect of 26H,7O-C on Hh signalling (25 μM 3β,5α-dihydroxycholest-7-en-6-one inhibited the effect of 10 μM 26H,7O-C in a Gli-luciferase assay by about 80 %) in a non-competitive manner, presumably through binding at a site on SMO distinct from the binding pocket populated by 26H,7O-C [86]. The concept of accessible cholesterol as a determinant of Hh signalling, as recently evoked by Kinnebrew et al. [82], defines only a small fraction of membrane cholesterol as accessible for Smo activation. It is not unreasonable to expect this pool of cholesterol to be reduced in SLOS, enhancing the importance of the balance between multiple competing oxysterol agonists of differing efficacy in determining the ultimate SLOS phenotype. Besides SLOS, other disorders of cholesterol biosynthesis and metabolism present with developmental malformations in tissues where embryonic patterning depends on Hh signalling [37]. Like SLOS these may also result from an imbalance of SMO agonists. Unlike, disorders of cholesterol biosynthesis, NP-C does not show dysmorphic features characteristic of SLOS nor do NP-C children show developmental delay. This suggests that the Hh signalling pathway is not disturbed in the NP-C foetus, presumably as the non-enzymatic formation of 7−OC, the initiating step in the 7-oxo/7β pathway in this disorder, is less prevalent in the foetus where partial pressure of oxygen is lower than after parturition. Further study will be required to test this hypothesis.

In summary, we have identified an unusual pathway of bile acid biosynthesis starting from 7-DHC, rather than cholesterol, which is evident in SLOS patients. The pathway is also active in healthy controls to a minor extent. The relative importance of pathway intermediates on Hh signalling may be revealed in future studies utilising sterol-specific mass spectrometry imaging in tissues where Hh signalling is required for development [20,85].

## 4 Experimental procedures

### 4.1 Materials

Sources of materials for LC–MS(MS^n^) analysis and of authentic standard compounds can be found in [32,[42], [43], [44],^49^].

### 4.2 Human samples

All participants or their parents/guardians provided informed consent and the study was performed with institutional review board approval (REC08/H1010/63) and adhered to the principles of the Declaration of Helsinki. Historical and newly collected SLOS plasma samples (n = 10 patients) were as described in [44]. SLOS urine samples (n = 3 patients) were from Central Manchester University Hospitals NHS Foundation Trust. The urine samples were form the group of patients who donated plasma. SLOS samples were received with limited clinical data i.e. sex, diagnosis. Deidentified amniotic fluid samples (SLOS affected pregnancies n = 5, healthy pregnancies n = 12) were from Kennedy Krieger Biochemical Genetics Laboratory, Baltimore, USA as reported in [47]. Control plasma samples (n = 24) were from a previously reported study [43]. Control urine samples (n = 6 volunteers) were kindly provided by the Joint Clinical Research Facility, Swansea University / ABMU Local Health Board.

### 4.3 Extraction and analysis of sterols and oxysterols from plasma and amniotic fluid

Sterols and oxysterols were extracted from plasma as described in [[42], [43], [44]]. The extraction procedure was essentially the same for amniotic fluid with minor modifications. We utilised a charge-tagging protocol to maximise sensitivity for LC–MS(MS^n^) analysis (Supplemental Figure S1). Full details are provided in Supplemental Experimental Procedures.

### 4.4 Extraction and analysis of bile acids from urine

Working solutions of [2,2,4,4-^2^H_4_]cholic acid (20 ng/μL), [2,2,4,4-^2^H_4_]glycochenodeoxycholic acid (20 ng/μL) and [2,2,4,4-^2^H_4_]taurochenodeoxycholic (20 ng/μL) were prepared in absolute ethanol. 2 μL (40 ng) of each working solution was added to 994 μL of water in a 2 mL microcentrifuge tube. Urine (100 μL, pH 6–7) was added drop-wise to the 1 mL of water containing deuterated standards. After 10 min ultrasonication the solution was centrifuged at 17,000 *g*, 4 °C for 30 min and the supernatant retained. An Oasis HLB (60 mg, Waters, Elstree, UK) column was washed with absolute ethanol (4 mL), methanol (4 mL) and conditioned with water (4 mL). The supernatant from above was loaded onto the column and allowed to flow at 0.25 mL/min. After a 3 mL wash with water, bile acids were eluted in 4 × 1 mL of methanol. The first two 1 mL fractions (containing bile acids) were combined, diluted to 60 % methanol and analysed by LC–MS(MS^n^) in an identical fashion to derivatised oxysterols [42] with the exception that bile acid urine analysis was performed in the negative ion mode.

### 4.5 Statistics

A Mann-Whitney test was used for non-normally distributed data. A P value of 0.05 or less was considered statistically significant.

### 4.6 Hedgehog signaling assays using quantitative RT-PCR

*Gli1* mRNA, encoded by a direct Hh target gene, was measured by quantitative real-time reverse-transcription PCR as described in [54] with minor modifications (see Supplemental Experimental Procedures).

### 4.7 Oxysterol ligand affinity chromatography

Purified zSmo ectodomain protein (zebrafish Smo CRD expressed in HEK-293 T cells) was diluted in 20 mM Tris pH 8.5, 150 mM NaCl, 0.3 % octyl-glucoside prior to addition of competitors (100 μM) and 20S−HC beads (1:200) [35]. Binding was allowed to proceed for 12 h at 4 °C, and then the resin was washed and captured protein eluted with four washes with sodium dodecyl sulfate (SDS) with 100 μM dithiothreitol (DTT). The zSmo ectodomain was measured by colloidal Coomassie staining [35].

### 4.8 Molecular docking

*In silico* protein-ligand docking studies were performed using a combination of empirical and force-field approaches incorporated within an in-house pipeline called “Shipyard”, to predict the conformation and affinity of binding. The crystal structure of the human Smo protein was obtained from the RCSB Protein Data Bank (PBD, 5L7D) in complex with cholesterol [52]. A mapping of all pockets and grooves of the Smo protein structure was generated using Sphgen [87]. The binding pockets were confirmed using DoGSiteScorer [88]. Output of the binding site search was verified manually via comparison with the original crystal structure. All ligand structures were subjected to the Open Babel system to make hydrogen atoms explicit [89].

To generate the 10 best poses for ligands in the Smo protein, docking was performed using DOCK 6 [87] and AutoDock Vina [53], which assign a binding energy score and rank. The pool of possible conformations was independently rescored using the DOCK 6 force field, the AutoDock Vina empirical scoring method, and the DSX (DrugScore eXtended) knowledge-based statistical potential [90]. An overall best pose was selected from the pool according to a consensus-by-rank scheme across the three scores. The AutoDock Vina score of the chosen conformation was taken as the prediction of the binding affinity of the ligand. Images of best poses of selected candidates were generated in Chimera [91].

## Footnote

The content is solely the responsibility of the authors and does not necessarily represent the official views of the National Institutes of Health.

## Declaration of Competing Interest

WJG, YW and PJC are listed as inventors on the patent “Kit and method for quantitative detection of steroids” US9851368B2. WJG, JAK and YW are listed as inventors on the patent application “Diagnostic methods and kits” WO2017037465A1. WJG, EY, PJC, JAK and YW are shareholders in CholesteniX Ltd.
