# Supplementary material for: Bile acid biosynthesis in Smith-Lemli-Opitz syndrome bypassing cholesterol: Potential importance of pathway intermediates
Source: J Steroid Biochem Mol Biol. 2021 Feb;206:105794. doi: 10.1016/j.jsbmb.2020.105794 (PMC7816163; doi:10.1016/j.jsbmb.2020.105794)
Supplement: Supplementary file 1 [file mmc1.docx]

**Bile Acid Biosynthesis in Smith-Lemli-Opitz Syndrome Bypassing Cholesterol: Potential Importance of Pathway Intermediates**

Jonas Abdel-Khalik^1^, Thomas Hearn^1^, Alison L. Dickson^1^, Peter J. Crick^1^, Eylan Yutuc^1^, Karl Austin-Muttitt^1^, Brian W. Bigger^2^, Andrew A. Morris^3^, Cedric H. Shackleton^4^, Peter T. Clayton^5^, Takashi Iida^6^, Ria Sircar^7^, Rajat Rohatgi^7^, Hanns-Ulrich Marschall^8^, Jan Sjövall^9^, Ingemar Björkhem^10^, Jonathan G.L. Mullins^1^, William J. Griffiths^1^, Yuqin Wang^1^

^1^Swansea University Medical School, ILS1 Building, Singleton Park, Swansea SA2 8PP, Wales, UK

^2^Stem Cell & Neurotherapies, Faculty of Biology, Medicine and Health, University of Manchester, Manchester M13 9PT, UK

^3^Willink Unit, Manchester Centre for Genomic Medicine, Manchester University Hospitals, Manchester M13 9WL, UK

^4^University of California San Francisco (UCSF) Benioff Children’s Hospital, Oakland, CA 94609, USA

^5^Inborn Errors of Metabolism, Genetics and Genomic Medicine, UCL Great Ormond Street Institute of Child Health, 30 Guilford Street, London WC1N 1EH, UK

^6^Department of Chemistry, College of Humanities & Sciences, Nihon University, Sakurajousui, Setagaya, Tokyo 156-8550, Japan

^7^Departments of Biochemistry and Medicine, Stanford University School of Medicine, Stanford, CA 94305, USA

^8^Department of Molecular and Clinical Medicine, University of Gothenburg, Sahlgrenska Academy, Institute of Medicine, Gothenburg 41345, Sweden

^9^Department of Medical Biochemistry and Biophysics, Karolinska Institutet, Stockholm 17177, Sweden

^10^Division of Clinical Chemistry, Department of Laboratory Medicine, Karolinska Institutet and Karolinska University Hospital Huddinge, Huddinge 14186, Stockholm, Sweden

**1. Supplemental Experimental Procedures**

*1.1. Extraction of Sterols and Oxysterols from Plasma*

Plasma (100 µL) was added dropwise to a solution of absolute ethanol (1.05 mL) containing [25,26,26,26,27,27,27-^2^H_7_]24R/S-hydroxycholesterol ([^2^H_7_]24-HC, Avanti Polar Lipids, Alabaster, AL) and [25,26,26,26,27,27,27-^2^H_7_]22R-hydroxycholest-4-en-3-one ([^2^H_7_]22R-HCO]) (20 ng of each) with sonication in an ultrasonic bath. After 5 min the solution was diluted to 70% ethanol by addition of 0.35 mL of water, ultrasonicated for a further 5 min and centrifuged at 17,000 × g at 4 ^o^C for 30 min. The supernatant was loaded onto a 200 mg Certified Sep-Pak C_18_ column (Waters, Elstree, Herts, UK), pre-conditioned with 4 mL of absolute ethanol followed by 6 mL 70% ethanol, and allowed to flow at ~0.25 mL/min. The flow-through was combined with a column wash of 70% ethanol (5.5 mL) to give SPE1-Fr1 containing the oxysterols and also C_24_ and C_27_ acids. A second fraction (SPE1-Fr2) was collected by eluting with a further 4 mL of 70% ethanol before elution of cholesterol, 7-DHC and similarly hydrophobic sterols using 2 mL of absolute ethanol (SPE1-Fr3). Each fraction was divided into two equal portions (A) and (B) and concentrated under reduced pressure using a vacuum concentrator (ScanLaf, Denmark).

*1.2. Charge-Tagging of Sterols and Oxysterols from Plasma*

We utilised a charge-tagging protocol to maximise sensitivity for LC-MS(MS^n^) analysis (Supplemental Figure S1) (1-6). The sterol and oxysterol fractions (A) from above were re-constituted in 100 µL of propan-2-ol then treated with KH_2_PO_4_ buffer (1 mL 50 mM, pH 7) containing 3 µL of cholesterol oxidase (2 mg/mL in H_2_O, 44 units/mg protein). The reaction mixture was incubated at 37 ^o^C for 1 hr then quenched with 2.0 mL of methanol. Glacial acetic acid (150 µL) was added followed by Girard P (GP) reagent ([^2^H_5_]GP 190 mg bromide salt or [^2^H_0_]GP 150 mg chloride salt, 0.80 mmol). The mixture was vortexed then incubated at room temperature overnight in the dark. To remove excess reagent from the reaction mixture a recycling method was used. A 200 mg Certified Sep-Pak C_18_ column was pre-conditioned with methanol (6 mL), 10% methanol (6 mL) and finally 70% methanol (4 mL). The derivatization mixture from above (3.25 mL in ~70% organic) was applied to the column and allowed to flow at a rate of ~0.25 mL/min. The column was washed with 70% methanol (1 mL) followed by 35% methanol (1 mL) and the combined eluent diluted with water (4 mL) to give a solution in 9 mL of 35% methanol. This solution was applied to the column, collected, and combined with a column wash of 17.5% methanol (1 mL). Water (9 mL) was added to give a solution in 19 mL of 17.5% methanol which was again applied to the column. The flow-through was discarded and the column washed with 10% methanol (6 mL). Derivatized sterols/oxysterols/acids were then eluted from the column with methanol (3 × 1 mL, SPE2-Fr1, -Fr2, -Fr3) followed by absolute ethanol (1 mL, SPE2-Fr4). Cholesterol and 7-DHC were found to be mostly present in SPE2-Fr3 while oxysterols/acids elute in SPE2-Fr1 and Fr2. The fractions (B) were treated in an identical fashion to the (A) fractions but in the absence of cholesterol oxidase. This allows differentiation of sterols oxidised to contain an oxo group from those naturally possessing one. Note, a saponification step was not carried out, so the molecules ultimately analysed were in their non-esterified form. In studies performed after this work the 200 mg Certified Sep-Pak C_18_ column (SPE2) has been replaced by an Oasis HLB 60 mg column (Waters, Elstree, Herts, UK) which shows less batch to batch variation (7).

*1.3. Extraction of Sterols and Oxysterols from Amniotic Fluid followed by Charge-Tagging*

The procedure for extraction of sterols and oxysterols from amniotic fluid was essentially that used for plasma with minor modification.

Amniotic fluid (100 µL) was subjected to single-phase extraction into acetonitrile (1.05 mL), rather than ethanol, and the [^2^H_7_]24-HC content was 7 ng rather than 20 ng. The extract was diluted to 70% acetonitrile (1.5 mL) and loaded onto the Sep-Pak C_18_ column (SPE1), the flow-through was combined with a column-wash of 70% ethanol (5.5 mL) to give SPE1-Fr1 (7 mL, 70% organic). The remainder of the extraction and charge-tagging protocol was identical to that used with plasma except the SPE2 column was Oasis HLB 60 mg.

*1.4. LC-MS(MS^n^) on the Hybrid Linear ion-trap (LIT)-Orbitrap*

To analyse GP-tagged oxysterols, including C_24_ and C_27_ acids, equal aliquots of SPE2-Fr1A, SPE2-Fr2A, SPE2-FR1B and SPE2-FR2B were combined then diluted to give a final solution of 60% methanol. For each experiment, 20 µL was injected onto the LC column and MS, MS^2^ and MS^3^ spectra recorded as described below. For the analysis of the more hydrophobic sterols equal volumes of SPE2-FR1A, SPE2-Fr2A, SPE2-Fr3A, SPE2-Fr1B, SPE2-Fr2B and SPE2-Fr3B were combined prior to dilution to 60% methanol.

LC was performed on an Ultimate 3000 LC system (Dionex, Surrey, UK, now Thermo Fischer) using a Hypersil GOLD reversed phase column (1.9 µm particle size, 50 × 2.1 mm, Thermo Fisher, Hemel Hempstead, UK). Mobile phase A consisted of 33.3% methanol, 16.7% acetonitrile, containing 0.1% formic acid. Mobile phase B consisted of 63.3% methanol, 31.7% acetonitrile, containing 0.1% formic acid. The chromatographic run started at 20% B for 1 min before increasing the proportion of B to 80% over 7 minutes and maintaining this for a further 5 min. The proportion of B was returned to 20% over 6 s and re-equilibration was for 3 min, 54 s to give a total run time of 17 min. The flow rate was 200 µL/min and the eluent was directed to the atmospheric pressure ionization (API) source of an LIT-Orbitrap mass spectrometer (LTQ-Orbitrap, Orbitrap Velos or Orbitrap Elite, Thermo Fisher, Hemel Hempstead, UK). The Orbitrap was calibrated externally before each analytical session and the mass accuracy was better than 5 ppm. The acquisition method consisted of a Fourier Transform (FT)-MS scan in the Orbitrap at 60,000 or 120,000 resolution (full width at half-maximum height; FWHM, at *m/z* 400), simultaneous to which sequential pre-defined MS^2^ and MS^3^ scans were carried out in the LIT with normalised collision energies of 30 for MS^2^ and 35 for MS^3^ (instrument settings).

*1.5. Hedgehog Signaling Assays Using Quantitative RT-PCR*

NIH/3T3 cells were grown to confluency in Dulbecco’s Modified Eagle’s Medium (DMEM) containing 10% Foetal Bovine Serum (FBS, Fisher Scientific, Loughborough, UK). Confluent cells were exchanged into 0.5% FBS DMEM for 24 hr to allow ciliogenesis prior to treatment with oxysterols (1 or 10 µM) in fresh DMEM containing 0.5% FBS for 7.5 hr. Oxysterols were added in ethanol vehicle to give a final solution of < 0.1% ethanol. RNA was extracted using a RNeasy mini kit (Qiagen, UK) and cDNA generated using a QuantiTect Reverse Transcription kit (Qiagen, UK). The mRNA levels of *Gli1*, a direct Hh target gene commonly used as a metric for signalling strength, were measured using the QuantiFast SYBR Green PCR kit (Qiagen, UK) (8). The primers used were *Gli1* (forward primer: 5’-ccaagccaactttatgtcaggg-3’ and reverse primer: 5’-agcccgcttctttgttaatttga-3’), *Rpl27* (forward primer: 5’- gtcgagatgggcaagttcat-3’ and reverse primer: 5’-gcttggcgatcttcttcttg-3’). Transcript levels relative to *Rpl27* were calculated using the ΔΔ-Ct method. Each qRT-PCR experiment was repeated with at least three technical replicates and two biological replicates.

**References**

1. Griffiths, W. J., P. J. Crick, Y. Wang, M. Ogundare, K. Tuschl, A. A. Morris, B. W. Bigger, P. T. Clayton, and Y. Wang. 2013. Analytical strategies for characterization of oxysterol lipidomes: liver X receptor ligands in plasma. *Free Radic Biol Med* **59**: 69-84.

2. Crick, P. J., T. William Bentley, J. Abdel-Khalik, I. Matthews, P. T. Clayton, A. A. Morris, B. W. Bigger, C. Zerbinati, L. Tritapepe, L. Iuliano, Y. Wang, and W. J. Griffiths. 2015. Quantitative charge-tags for sterol and oxysterol analysis. *Clin Chem* **61**: 400-411.

3. Griffiths, W. J., E. Yutuc, J. Abdel-Khalik, P. J. Crick, T. Hearn, A. Dickson, B. W. Bigger, T. Hoi-Yee Wu, A. Goenka, A. Ghosh, S. A. Jones, D. F. Covey, D. S. Ory, and Y. Wang. 2019. Metabolism of Non-Enzymatically Derived Oxysterols: Clues from sterol metabolic disorders. *Free Radic Biol Med* **144**: 124-133.

4. Griffiths, W. J., I. Gilmore, E. Yutuc, J. Abdel-Khalik, P. J. Crick, T. Hearn, A. Dickson, B. W. Bigger, T. H. Wu, A. Goenka, A. Ghosh, S. A. Jones, and Y. Wang. 2018. Identification of unusual oxysterols and bile acids with 7-oxo or 3beta,5alpha,6beta-trihydroxy functions in human plasma by charge-tagging mass spectrometry with multistage fragmentation. *J Lipid Res* **59**: 1058-1070.

5. Abdel-Khalik, J., E. Yutuc, P. J. Crick, J. A. Gustafsson, M. Warner, G. Roman, K. Talbot, E. Gray, W. J. Griffiths, M. R. Turner, and Y. Wang. 2017. Defective cholesterol metabolism in amyotrophic lateral sclerosis. *J Lipid Res* **58**: 267-278.

6. Griffiths, W. J., P. J. Crick, and Y. Wang. 2013. Methods for oxysterol analysis: past, present and future. *Biochem Pharmacol* **86**: 3-14.

7. Crick, P. J., T. W. Bentley, Y. Wang, and W. J. Griffiths. 2015. Revised sample preparation for the analysis of oxysterols by enzyme-assisted derivatisation for sterol analysis (EADSA). *Anal Bioanal Chem* **407**: 5235-5239.

8. Luchetti, G., R. Sircar, J. H. Kong, S. Nachtergaele, A. Sagner, E. F. Byrne, D. F. Covey, C. Siebold, and R. Rohatgi. 2016. Cholesterol activates the G-protein coupled receptor Smoothened to promote Hedgehog signaling. *Elife* **5**.
