# Supplementary material for: Bile acid biosynthesis in Smith-Lemli-Opitz syndrome bypassing cholesterol: Potential importance of pathway intermediates
Source: J Steroid Biochem Mol Biol. 2021 Feb;206:105794. doi: 10.1016/j.jsbmb.2020.105794 (PMC7816163; doi:10.1016/j.jsbmb.2020.105794)
Supplement: Supplementary file 2 [file mmc2.pdf]

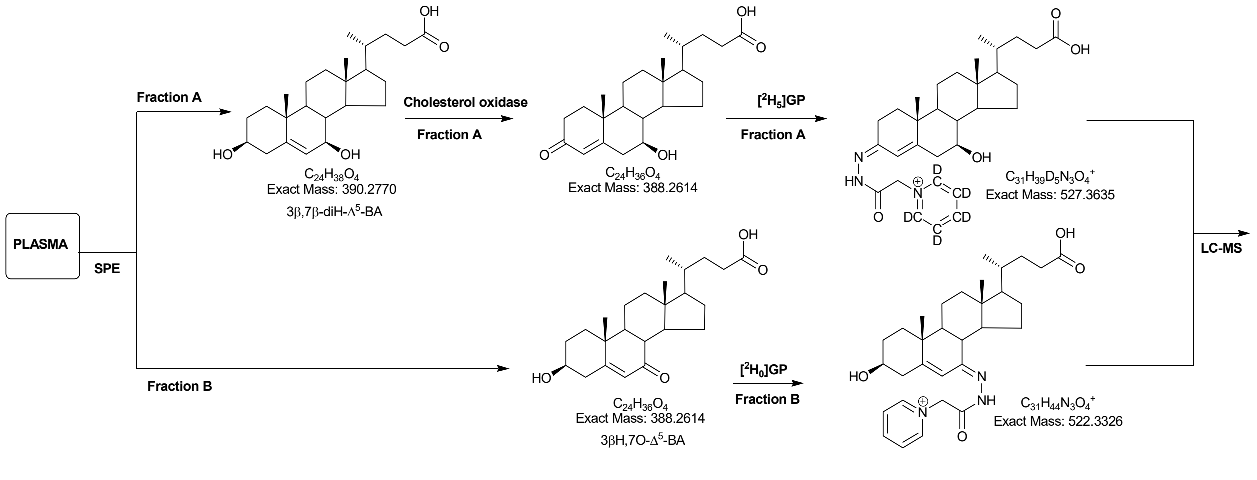

Figure S1. Principles of charge-tagging exemplified for 3β,7β-diH-Δ^5-BA and 3βH,7O-Δ^5-BA.

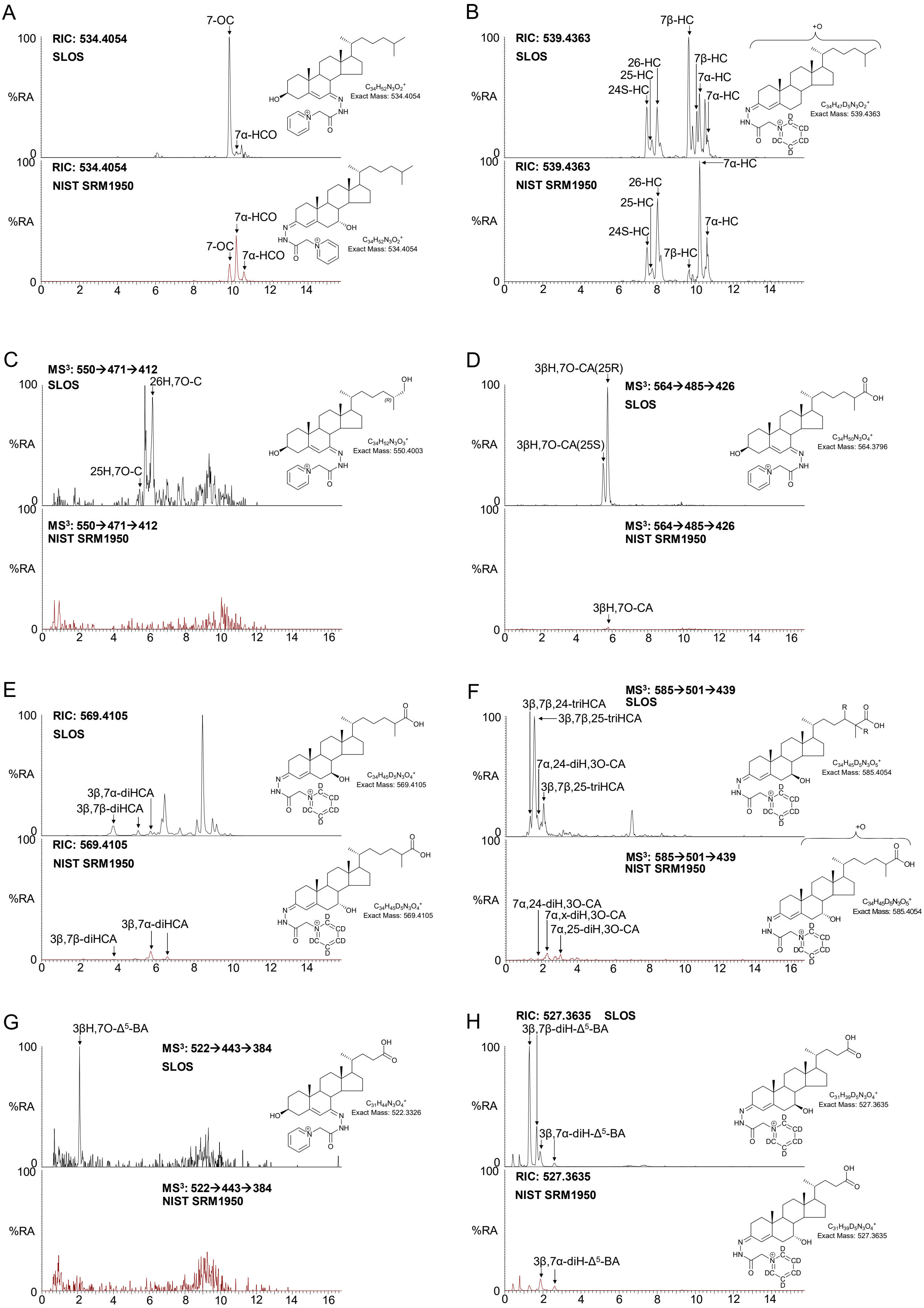
A
RIC: 534.4054
SLOS
7-OC
7α-HCO
RIC: 534.4054
NIST SRM1950
7α-HCO
7-OC
7α-HCO
B
RIC: 539.4363
SLOS
7β-HC
26-HC
25-HC
24S-HC
7β-HC
7α-HC
7α-HC
RIC: 539.4363
NIST SRM1950
7α-HC
26-HC
25-HC
24S-HC
7β-HC
7α-HC
C
MS³: 550→471→412
SLOS
26H,7O-C
25H,7O-C
MS³: 550→471→412
NIST SRM1950
D
3βH,7O-CA(25R)
MS³: 564→485→426
SLOS
3βH,7O-CA(25S)
MS³: 564→485→426
NIST SRM1950
3βH,7O-CA
E
RIC: 569.4105
SLOS
3β,7α-diHCA
3β,7β-diHCA
RIC: 569.4105
NIST SRM1950
3β,7α-diHCA
3β,7β-diHCA
F
MS³: 585→501→439
SLOS
3β,7β,24-triHCA
3β,7β,25-triHCA
7α,24-diH,3O-CA
3β,7β,25-triHCA
MS³: 585→501→439
NIST SRM1950
7α,24-diH,3O-CA
7α,x-diH,3O-CA
7α,25-diH,3O-CA
G
3βH,7O-Δ⁵-BA
MS³: 522→443→384
SLOS
MS³: 522→443→384
NIST SRM1950
H
RIC: 527.3635 SLOS
3β,7β-diH-Δ⁵-BA
3β,7α-diH-Δ⁵-BA
RIC: 527.3635
NIST SRM1950
3β,7α-diH-Δ⁵-BA
%RA
Exact Mass: 534.4054
Exact Mass: 539.4363
Exact Mass: 550.4003
Exact Mass: 564.3796
Exact Mass: 569.4105
Exact Mass: 585.4054
Exact Mass: 522.3326
Exact Mass: 527.3635

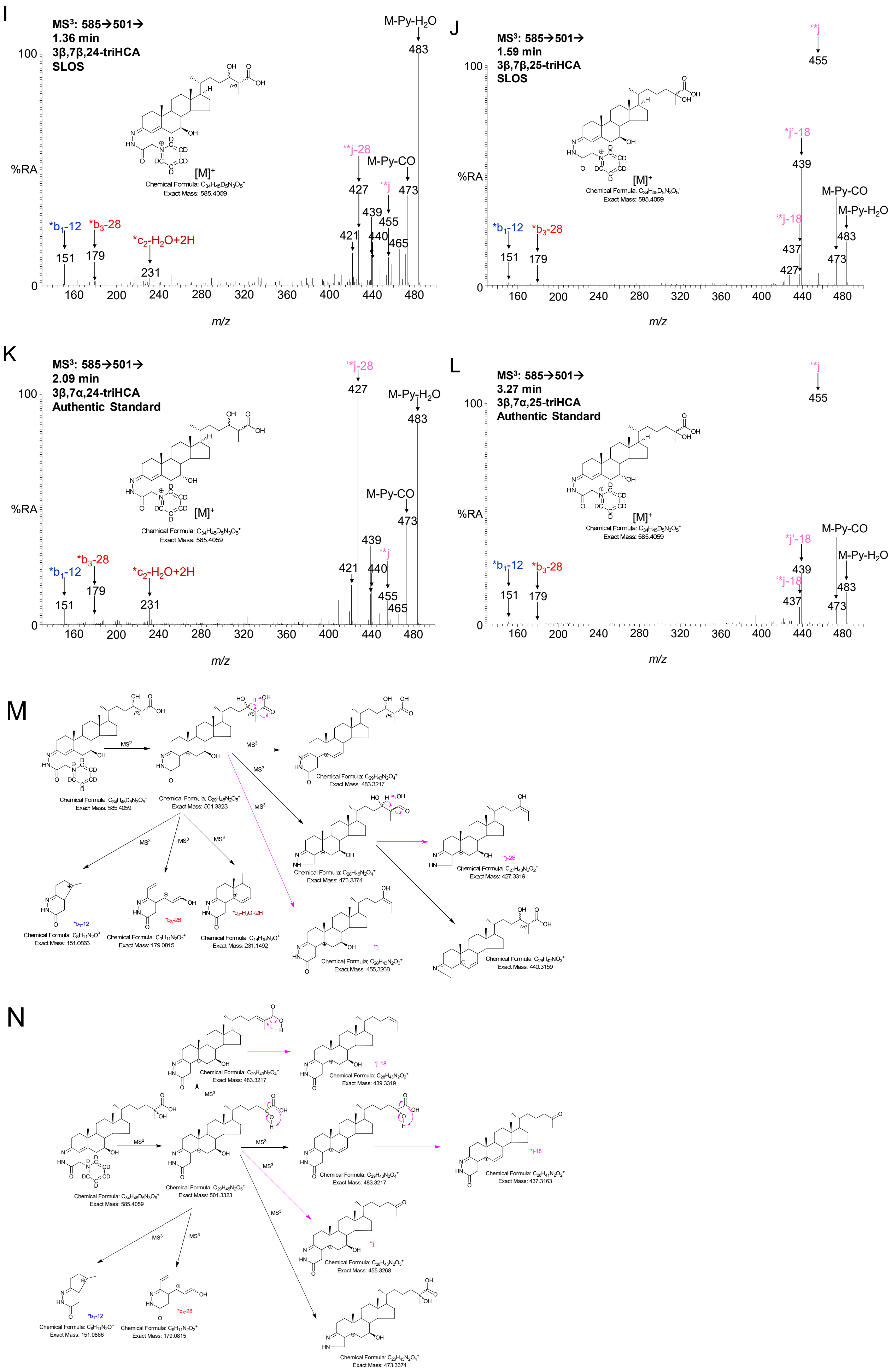
I
MS3: 585→501→
1.36 min
3β,7β,24-triHCA
SLOS
M-Py-H2O
483
‘*j-28
M-Py-CO
427
473
‘*j
439
455
421
440
465
*b1-12
*b3-28
*c2-H2O+2H
151
179
231
%RA
m/z
[M]+
Chemical Formula: C34H45D5N3O5+
Exact Mass: 585.4059
J
MS3: 585→501→
1.59 min
3β,7β,25-triHCA
SLOS
‘*j
455
*j'-18
439
M-Py-CO
M-Py-H2O
‘*j-18
483
437
427
473
*b1-12
*b3-28
151
179
K
MS3: 585→501→
2.09 min
3β,7α,24-triHCA
Authentic Standard
‘*j-28
427
M-Py-H2O
483
M-Py-CO
473
439
421
440
455
465
*b1-12
*b3-28
*c2-H2O+2H
151
179
231
L
MS3: 585→501→
3.27 min
3β,7α,25-triHCA
Authentic Standard
‘*j
455
M-Py-CO
*j'-18
M-Py-H2O
439
‘*j-18
483
437
473
*b1-12
*b3-28
151
179
M
MS2
MS3
Chemical Formula: C34H45D5N3O5+
Exact Mass: 585.4059
Chemical Formula: C29H45N2O5+
Exact Mass: 501.3323
Chemical Formula: C29H43N2O4+
Exact Mass: 483.3217
Chemical Formula: C28H45N2O4+
Exact Mass: 473.3374
‘*j-28
Chemical Formula: C27H43N2O2+
Exact Mass: 427.3319
‘*j
Chemical Formula: C28H43N2O3+
Exact Mass: 455.3268
Chemical Formula: C28H42NO3+
Exact Mass: 440.3159
*b1-12
Chemical Formula: C9H11N2O+
Exact Mass: 151.0866
*b3-28
Chemical Formula: C9H11N2O2+
Exact Mass: 179.0815
*c2-H2O+2H
Chemical Formula: C14H19N2O+
Exact Mass: 231.1492
N
Chemical Formula: C29H43N2O4+
Exact Mass: 483.3217
*j'-18
Chemical Formula: C28H43N2O2+
Exact Mass: 439.3319
Chemical Formula: C34H45D5N3O5+
Exact Mass: 585.4059
Chemical Formula: C29H45N2O5+
Exact Mass: 501.3323
‘*j-18
Chemical Formula: C28H41N2O2+
Exact Mass: 437.3163
‘*j
Chemical Formula: C28H43N2O3+
Exact Mass: 455.3268
*b1-12
Chemical Formula: C9H11N2O+
Exact Mass: 151.0866
*b3-28
Chemical Formula: C9H11N2O2+
Exact Mass: 179.0815
Chemical Formula: C28H45N2O4+
Exact Mass: 473.3374

Figure S2. LC-MS(MS^n) analysis of oxysterols, including C_{24} and C_{27} acids, in plasma. Data was generated on a hybrid LIT-Orbitrap. Samples prepared in the presence of cholesterol oxidase utilised the [$^2H_5$]GP derivatisation reagent resulting in odd *m/z* molecular ions, samples prepared in the absence of cholesterol oxidase utilised [$^2H_0$]GP reagent resulting in even *m/z* molecular ions. Upper panels are for an SLOS plasma sample, lower panels are for the NIST SRM 1950 (1). Upper and lower panels are shown on the same y-axis. GP derivatives give *syn* and *anti* conformers, which may or may not be resolved. (A) Reconstructed ion chromatogram (RIC) of *m/z* 534.4054 ± 10 ppm corresponding to 7-OC and 7α-hydroxycholest-4-en-3-one (7α-HCO). Samples were prepared in the absence of cholesterol oxidase. (B) RIC of *m/z* 539.4363 ± 10 ppm corresponding to monohydroxycholesterols (HC). Samples were prepared in the presence of cholesterol oxidase. (C) MS^3 multiple reaction monitoring transition (MRM) 550→471→412 characteristic of 26H,7O-C. Samples were prepared in the absence of cholesterol oxidase. (D) MS^3 MRM 564→485→426 characteristic of 3βH,7O-CA. Note, both 25R and 25S-epimers are resolved. Samples were prepared in the absence of cholesterol oxidase. (E) RIC of *m/z* 569.4105 ± 10 ppm corresponding to dihydroxycholestenoic acids (diHCA). Samples were prepared in the presence of cholesterol oxidase. (F) MS^3 MRM 585→501→439 characteristic of the trihydroxycholestenoic acids (triHCA) 3β,7,24(or25)-triHCA. Samples were prepared in the presence of cholesterol oxidase. (G) MS^3 MRM 522→443→384 characteristic of 3βH,7O-Δ^5-BA. Samples were prepared in the absence of cholesterol oxidase. (H) RIC of *m/z* 527.3635 ± 10 ppm corresponding to dihydroxycholenoic (diH-Δ^5-BA) and hydroxyoxocholenoic acids. Samples were prepared in the presence of cholesterol oxidase. MS^3 ($[M]^+$→$[M\text{-}Py]^+$→) spectra of (I) 3β,7β,24-triHCA, (J) 3β,7β,25-triHCA, both from SLOS plasma, and authentic standards (K) 3β,7α,24-triHCA and (L) 3β,7α,25-triHCA. Fragmentation patterns of (M) 3β,7β,24-triHCA and (N) 3β,7β,25-triHCA. Details of the fragmentation pathways of 7-oxo compounds in (C), (D) and (G) can be found in reference (2).

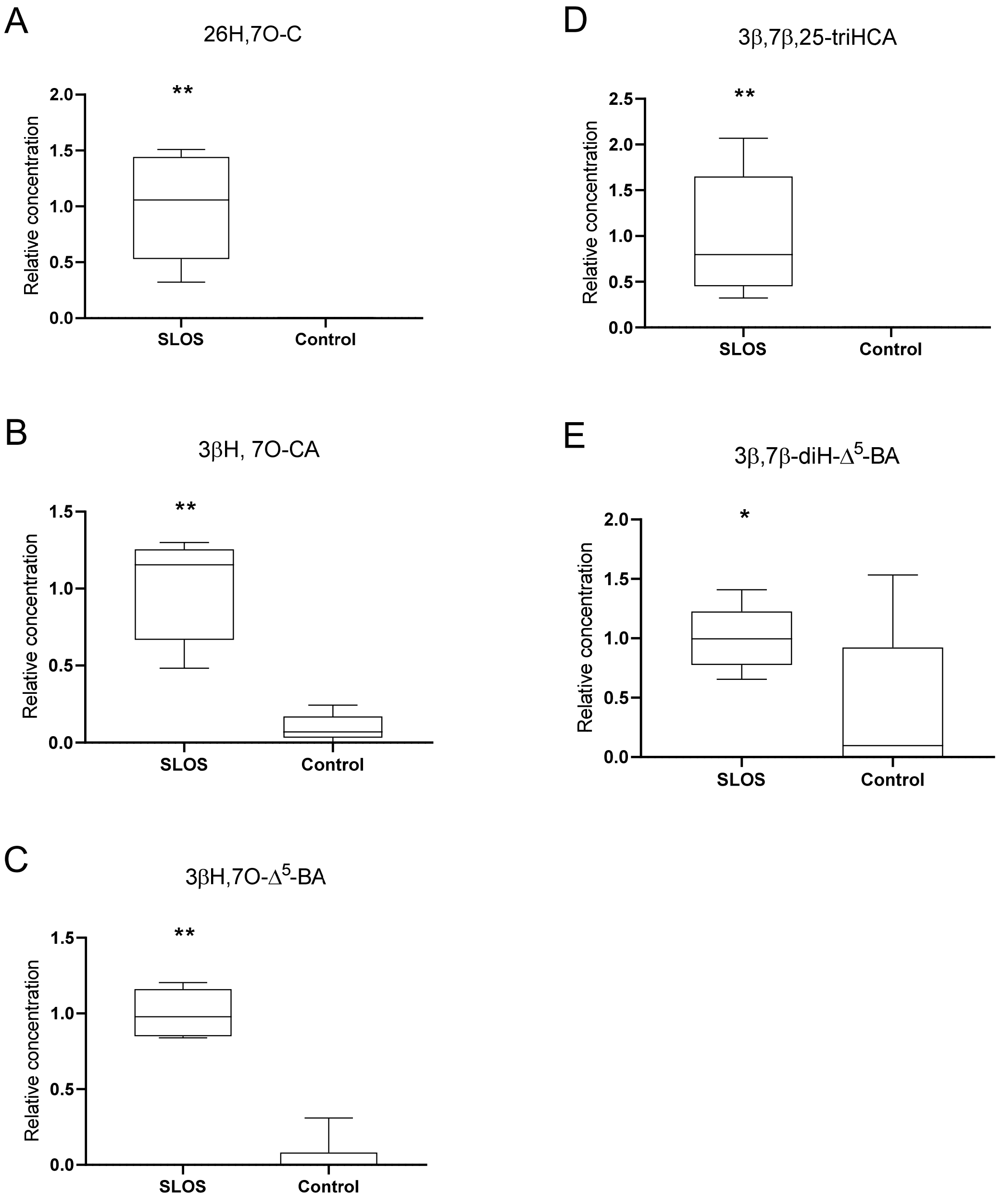

Figure S3. Relative concentrations of 26-hydroxy-7-oxocholesterol and 7β-hydroxy- and 7-oxo- C_{24} and C_{27} acids in amniotic fluid from SLOS affected pregnancies (n = 5) and healthy pregnancies (n = 12). (A) 26H,7O-C. (B) 3βH,7O-CA. (C) 3βH,7O-Δ^5-BA. (D) 3β,7β,25-triHCA. (E) 3β,7β-diH-Δ^5-BA. For each analyte relative concentrations were determined against [$^2H_7$]24R/S-HC internal standard and normalised to the mean value for SLOS set to 1. Statistical comparisons are made as described in the caption to Figure 2.

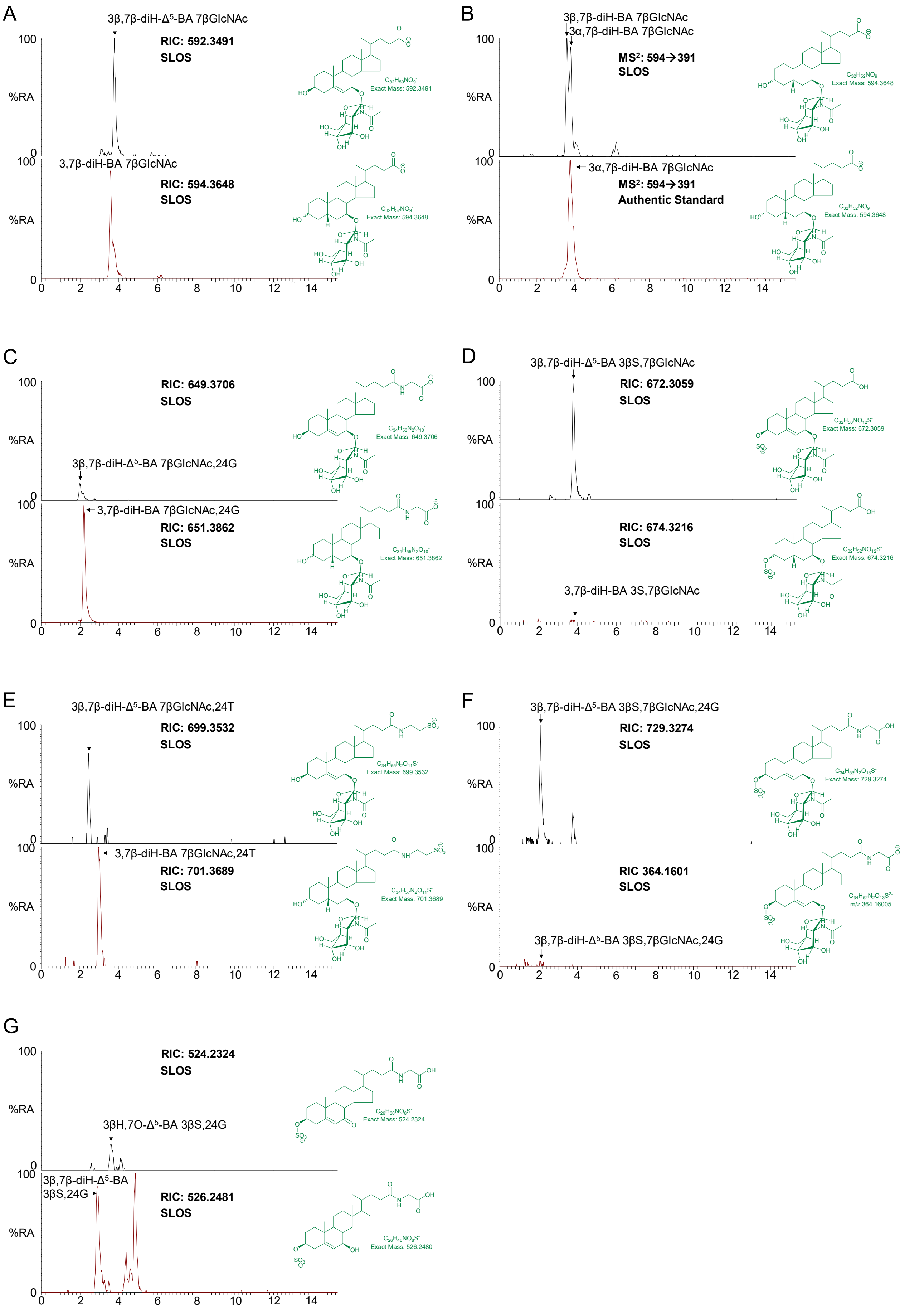
A
3β,7β-diH-Δ⁵-BA 7βGlcNAc
RIC: 592.3491
SLOS
3,7β-diH-BA 7βGlcNAc
RIC: 594.3648
SLOS
B
3β,7β-diH-BA 7βGlcNAc
3α,7β-diH-BA 7βGlcNAc
MS²: 594→391
SLOS
3α,7β-diH-BA 7βGlcNAc
MS²: 594→391
Authentic Standard
C
RIC: 649.3706
SLOS
3β,7β-diH-Δ⁵-BA 7βGlcNAc,24G
3,7β-diH-BA 7βGlcNAc,24G
RIC: 651.3862
SLOS
D
3β,7β-diH-Δ⁵-BA 3βS,7βGlcNAc
RIC: 672.3059
SLOS
RIC: 674.3216
SLOS
3,7β-diH-BA 3S,7βGlcNAc
E
3β,7β-diH-Δ⁵-BA 7βGlcNAc,24T
RIC: 699.3532
SLOS
3,7β-diH-BA 7βGlcNAc,24T
RIC: 701.3689
SLOS
F
3β,7β-diH-Δ⁵-BA 3βS,7βGlcNAc,24G
RIC: 729.3274
SLOS
RIC 364.1601
SLOS
3β,7β-diH-Δ⁵-BA 3βS,7βGlcNAc,24G
G
RIC: 524.2324
SLOS
3βH,7O-Δ⁵-BA 3βS,24G
3β,7β-diH-Δ⁵-BA
3βS,24G
RIC: 526.2481
SLOS
%RA
100
0
0 2 4 6 8 10 12 14

Figure S4. LC-MS(MS^n) analysis of GlcNAc conjugated bile acids in SLOS urine. Data was generated on a hybrid LIT-Orbitrap in the negative-ion mode. RIC ± 10 ppm are of $[M-H]^-$ ions (unless stated otherwise). Upper and lower panels are shown on the same y-axis. (A) RIC of *m/z* 592.3491 (upper panel) and 594.3648 (lower panel) corresponding to 3β,7β-diH-Δ^5-BA 7βGlcNAc and 3,7β-diH-BA 7βGlcNAc, respectively. (B) MS^2 MRM 594→391 characteristic of 3,7β-diH-BA 7βGlcNAc. The upper panel shows partial separation of 3α,7β-diH-BA 7βGlcNAc from the 3β-epimer in an SLOS sample. The lower panel shows 3α,7β-diH-BA 7βGlcNAc authentic standard. (C) RIC of *m/z* 649.3706 (upper panel) and *m/z* 651.3862 (lower panel) corresponding to 3β,7β-diH-Δ^5-BA 7βGlcNAc,24G and 3,7β-diH-BA 7βGlcNAc,24G, respectively. (D) RIC of *m/z* 672.3059 (upper panel) and *m/z* 674.3216 (lower panel) corresponding to 3β,7β-diH-Δ^5-BA 3βS,7βGlcNAc and 3,7β-diH-BA 3S,7βGlcNAc, respectively. (E) RIC of *m/z* 699.3532 (upper panel) and *m/z* 701.3689 (lower panel) corresponding to 3β,7β-diH-Δ^5-BA 7βGlcNAc,24T and 3,7β-diH-BA 7βGlcNAc,24T, respectively. (F) RIC of *m/z* 729.3274 (upper panel) and *m/z* 364.1601 (lower panel) corresponding to 3β,7β-diH-Δ^5-BA 3βS,7βGlcNAc,24G as $[M-H]^-$ and $[M-2H]^{2-}$ ions, respectively. (G) RIC of *m/z* 524.2324 (upper panel) and *m/z* 526.2481 (lower panel) corresponding to 3βH,7O-Δ^5-BA 3βS,24G and 3β,7β-diH-Δ^5-BA 3βS,24G, respectively.

1. Quehenberger, O., A. M. Armando, A. H. Brown, S. B. Milne, D. S. Myers, A. H. Merrill, S. Bandyopadhyay, K. N. Jones, S. Kelly, R. L. Shaner, C. M. Sullards, E. Wang, R. C. Murphy, R. M. Barkley, T. J. Leiker, C. R. Raetz, Z. Guan, G. M. Laird, D. A. Six, D. W. Russell, J. G. McDonald, S. Subramaniam, E. Fahy, and E. A. Dennis. 2010. Lipidomics reveals a remarkable diversity of lipids in human plasma. *J Lipid Res* **51**: 3299-3305.
2. Griffiths, W. J., I. Gilmore, E. Yutuc, J. Abdel-Khalik, P. J. Crick, T. Hearn, A. Dickson, B. W. Bigger, T. H. Wu, A. Goenka, A. Ghosh, S. A. Jones, and Y. Wang. 2018. Identification of unusual oxysterols and bile acids with 7-oxo or 3beta,5alpha,6beta-trihydroxy functions in human plasma by charge-tagging mass spectrometry with multistage fragmentation. *J Lipid Res* **59**: 1058-1070.
